# Decoding *cis*-regulatory elements in the germline of the human malaria vector *Anopheles gambiae*

**DOI:** 10.1038/s42003-026-10117-y

**Published:** 2026-05-02

**Authors:** Emily Chesters, Lara Ravenscroft, Trevor A. Thompson, Lakamy Sylla, Boubacar Tembely, Jak Kerr, Jacob Hasenauer, Nicole Page, Teena Winny, Tony Nolan, Igor Sharakhov, Daniel Tonge, Roberto Galizi

**Affiliations:** 1 Centre for Applied Entomology and Parasitology, School of Life Sciences, Keele University, Keele, UK; 2 Infectious Diseases and Medical Entomology Research and Training Center, Université des Sciences, des Techniques et des Technologies de Bamako, Bamako, Mali; 3 Department of Vector Biology, Liverpool School of Tropical Medicine, Liverpool, UK; 4 Department of Entomology, Virginia Polytechnic Institute and State University, Blacksburg, VA USA

**Keywords:** Gene regulation, Malaria

## Abstract

*Cis*-regulatory elements (CREs) drive tissue- and cell-specific gene expression and are essential for safe, sustainable genetic control strategies in pest and vector insects, including the engineering of gene drives in the primary human-malaria vector *Anopheles gambiae*. Yet CREs remain poorly defined in mosquitoes due to limited computational tools and practical methods for identification and validation. We present a systematic in silico approach for CRE discovery, correlating targeted DNA-motif searches with gene expression, followed by frequency and distribution analysis within putative promoter regions. Applied to the *A. gambiae* germline, this approach identified hundreds of putative CREs significantly correlated with germline expression in one or both sexes, often linked to distinct sperm developmental stages and chromosomal locations, suggesting roles in broader regulatory mechanisms such as dosage compensation and meiotic silencing. When mapped onto pre-characterised germline promoters, CRE distribution aligned with regions associated with experimental expression patterns. Finally, we validated a top-ranked testis-enriched CRE using an in vivo dual-reporter assay, showing that mutation of conserved nucleotides drastically altered male germline expression. To the best of our knowledge this work provides the first nucleotide-resolution regulatory genome annotation of the *A. gambiae* germline, offering a transferable framework to aid promoter design for genetic control strategies against malaria mosquitoes and other insect pests.

## Abstract (web summary)

Nucleotide-resolution mappings of germline *cis*-regulatory elements in the human malaria vector *Anopheles gambiae* provides a blueprint for improved tissue-specific engineering to advance genetic control of vector-borne diseases and insect pests.

## Introduction

*C is*-regulatory elements (CREs) such as transcription factor binding sites (TFBSs) are canonically located in promoter regions near transcription start sites (TSSs) acting as starting blocks for the recruitment of the transcriptional machinery required for gene expression. Knowledge of CREs responsible for tissue- and cell-type-specific gene activation is crucial for the engineering of genetic control strategies against vector and pest insects such as *Anopheles gambiae*, a major mosquito vector of human malaria which remains a global health burden. For example, homing gene drives require endonuclease expression to be restricted to diploid germline cells to initiate homology-directed repair (HDR) upon cleavage of DNA^1–4^. However, current efforts are hampered by leaky expression of the endonuclease in somatic cells or endonuclease activity into embryos post-fertilisation, both of which favour repair via error-prone non-homologous end-joining (NHEJ) or microhomology-mediated end-joining (MMEJ) pathways. These mechanisms generate resistant alleles that reduce fitness of transgenic insects, and compromise effectiveness of the drive due to selection pressures^2,3,5–8^. Both leaky expression and resistant allele generation derive from inadequate understanding of CREs in these insects, which reflects the current lack of suitable promoter elements to restrict gene editing activity to defined cell types.

In *Anopheles* mosquitoes, only a few promoters have been tested in homing gene drives to varying degrees of success, mostly limited to *A. gambiae*^3,4,6^ and *A. stephensi* species^2^. First generation gene drives were engineered using the *vasa2* promoter, which drives expression in both male and female germlines^9^. However, effectiveness was dramatically reduced due to Cas9-gRNA ribonucleoprotein (RNP) deposition in embryos and/or leaky expression in somatic cells^2,3,6^. To date, only the *zpg* promoter, a −1074 bp intergenic region upstream of the endogenous gene, has been successfully used to engineer effective gene drive systems in *A. gambiae* although showing imperfect efficiency likely due to parental carryover of the RNP complex leading to reduced fitness in drive-carrying heterozygous females^4,6^. A limited number of alternative germline promoters, including *nos* and *exu*, have also been explored for the development of homing gene drives in *Anopheles*, albeit with comparatively lower efficacy^6^. Despite the recent development of high resolution germline transcriptomes in the major insect vectors^10–12^, the current lack of CRE knowledge in mosquitoes impedes progress in the identification of suitable promoter elements or in defining their core regulatory regions.

Similarly, sex-ratio distortion strategies rely on endonuclease activity confined to male meiosis to selectively disrupt the X chromosome in haploid germline cells, favouring Y-bearing sperm fertilisation thus producing a male-biased progeny^13–15^. The transformation of this approach into a gene drive system (termed Y-drive)^16^, is still hindered by the lack of suitable promoters to express Y-linked transgenes during meiosis due to a widely conserved biological process known as meiotic sex chromosome inactivation (MSCI), also described in *Anopheles*^11,12^. From the limited set of germline promoters currently available in *A. gambiae*, the *β2-tubulin* promoter is the only male-specific meiotic promoter characterised so far^17^ and proved unable to express transgenes when located on the Y or X chromosome^13,18,19^. This highlights the need to expand knowledge of CRE roles in epigenetic processes of gene regulation, such as MSCI and dosage compensation, to pinpoint those that may either have a function in transcriptional repression in the germline^20–22^ or that could allow expression at defined meiotic developmental stages preceding MSCI^23^.

Overall, performance of the few germline promoters used to engineer genetic control technologies varies across insect species^24–28^, and efforts to translate gene drive systems to other medically and agriculturally relevant species have proven difficult so far. For example, in the major arboviral disease vector *Aedes aegypti*, regions −2 kb and −4 kb upstream of the orthologue *zpg* coding sequence failed to induce substantial biased gene drive inheritance, while only a small subset of other germline promoters showed moderate success^24,29^. At the heart of these problems lies an incomplete understanding of gene regulation, particularly in non-model insects for which promoter regions, commonly used to express transgenes such as CRISPR-Cas endonucleases, are usually described as the ~2 kb intergenic region upstream of the corresponding gene start codon^6,30–32^. This approach is prone to the inclusion of irrelevant genetic regions or CREs from overlapping genes that may have undesirable effects on gene expression. Similar challenges reflect a limited understanding of CRE function required for the development of human gene therapies, where the lack of suitable tissue-specific promoters has led to reliance on ubiquitous promoters, which can lead to unintended effects and compromise both safety and efficacy^33–36^. The limited understanding of CREs and gene regulation in insects is further complicated due to the paucity of omics data and regulatory genome annotations with adequate cell-type resolution^11,37–39^. Early efforts to identify CREs in *Anopheles* relied on comparative genomics, using phylogenetic footprinting to detect conserved motifs across species^40^ or leveraging open-chromatin profiling to guide motif discovery. For example, accessible chromatin regions in the midgut and salivary glands have been used to infer promoter-associated motifs^41,42^, and to identify putative DNA-binding sites of proteins involved in sex-specific regulation and dosage compensation^43–45^. Despite these advances, applying such approaches to promoter design in non-model insects remains challenging due to the lower resolution of comparative datasets, sparse genome annotations, and the lack of suitable methods for in vivo functional validation. Developing approaches that could infer CREs from existing datasets can therefore provide new routes to overcome these limitations with minimal resource needs. Here, we present a systematic framework to identify and characterise CREs using publicly accessible datasets that we apply to improve understanding of regulatory elements in the *A. gambiae* germline thus supporting the rational design of genetic control strategies both in mosquito vectors and other pest species.

## Results

### Computational motif discovery and analysis reveal putative *cis*-regulatory elements correlated with *Anopheles gambiae* germline expression

To address the current lack of suitable methods to identify and characterise CREs in insects, we developed an in silico approach for the identification and characterisation of putative CREs associated with gene expression. We validated our approach on *A. gambiae* germline-specific genes due to their importance for the development of genetic control strategies.

**Fig. 1:**
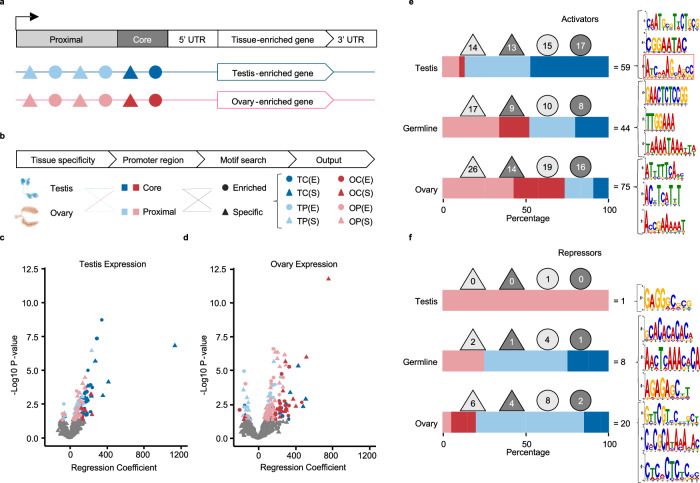
Putative CREs correlated with gene activation and/or repression in the *A. gambiae* testis and ovary. **a** Schematic of core (dark grey) and proximal (light grey) promoter regions used in the analysis. **b** Schematic of the in silico pipeline used, with dark red and blue referring to core promoter motifs and light red and blue indicating proximal promoter motifs, ‘enriched’ motifs (circle) refer to those that originate from searches with no specified control sequences, and ‘specific’ motifs (triangle) refer to those from searches including control sequences. **c**, **d** Volcano plots of linear regression analysis correlating the gene expression in the testis and ovary with presence of motifs originating from: testis-enriched (TC, TP) and ovary-enriched (OC, OP) gene sets and testis (**c**) or ovary expression (**d**). Coloured points represent motifs with linear regression *p* ≤ 0.05 in the respective tissue, whilst grey points are motifs that did not meet this threshold. **e**, **f** Stacked bar plots representing the origin of motifs (TC, TP, OC, and OP) showing significant correlation with gene (**e**) activation or (**f**) repression in the ‘testis’, ‘ovary’, and both tissues (referred to as ‘germline’). Circles (enriched) and triangles (specific) represent distribution of motifs found from each search, with light-grey triangles representing proximal specific, dark-grey triangles representing core specific, light-grey circles representing proximal enriched, and dark-grey circles representing core enriched. DNA motif logos represent the top three candidate motifs for each category (only one testis repressor was identified) based on regression significance.

First, we used the most comprehensive tissue-specific expression dataset for *A. gambiae*^46^ to select 100 genes with testis-enriched expression and 100 genes with ovary-enriched expression—herein referred to as ‘testis genes’, ‘ovary genes’, or ‘germline genes’ collectively (Fig. 1a). We also retrieved an equal number of male and female genes with low enrichment and expression in the testis and ovary—herein referred to as ‘male control genes’, ‘female control genes’, and ‘control genes’ collectively—as the corresponding control sets (Supplementary Data 1). We performed motif discovery to search for overrepresented motifs in the putative core (−100 bp to +30 bp from the TSS) and proximal (−500 bp to −101 bp from the TSS) promoter regions of each gene group. Motifs of 8–15 bp width were retrieved from the putative core and proximal promoter regions of testis and ovary gene sets either i) using a matched number of control sequences (male and female controls) from the respective sex as the specified background sequences—herein defined as ‘testis, ovary or germline-specific motifs’—or ii) without specified control sequences to capture ‘testis, ovary or germline-enriched motifs’. In parallel, we searched the core and proximal regions of control gene promoters, using the germline promoters from the corresponding sex as controls or shuffled sequences as background—herein referred to as ‘male/female control-specific motifs’ and ‘enriched motifs’. We imposed no significance thresholds in the motif searches to allow selection of low-frequency motifs which may occur only in subsets of genes (e.g. specific to a defined cell-type cluster within the tissue), meanwhile allowing a relatively small number of input sequences for each group to be used as input. Each search (specific or enriched in core or proximal sequences) was restricted to identify a maximum of 100 motifs, resulting in at most 400 motifs for each gene set that were categorised based on the origin (testis, ovary, male or female control), and promoter region (core or proximal) as follows: ‘testis core’ (TC), ‘ovary core’ (OC), ‘male control core’ (MCC), ‘female control core’ (FCC), ‘testis proximal’ (TP), ‘ovary proximal’ (OP), ‘male control proximal’ (MCP), and ‘female control proximal’ (FCP) motifs (Fig. 1b). We then measured the motif counts within the respective core and proximal promoter regions of all *A. gambiae* genes with annotated 5’ UTR and available tissue expression data (*n* = 8735) and performed standard linear regression analysis to correlate individual motif presence with testis or ovary gene expression. Outputs from the enriched and specific searches in germline and control genes were respectively combined to ease downstream analysis (Fig. 1; Supplementary Data 2).

Out of 771 germline-derived motifs, 207 showed significant correlation (*p* ≤ 0.05) with testis and/or ovary expression (Supplementary Data 2). The majority (178/207) had positive regression coefficients and were categorised as putative ‘activators’, whereas a smaller proportion (29/207) had negative regression coefficients and were defined as putative ‘repressors’ (Fig. 1c–f, Supplementary Data 2). The biased identification of activators (two-sided exact binomial test vs. 50%: *p* = 2.4 × 10^−27^) reflects the germline biased expression of the gene promoters used as respective inputs. Among the 178 activators, 59 positively correlated exclusively with testis gene expression, 75 exclusively with ovary, and 44 with testis and ovary expression herein respectively referred to as ‘testis, ovary or germline activators’ (Fig. 1e). Of the 29 repressors, only 1 showed inverse correlation exclusively with testis expression, 20 with ovary, and 8 with both germline tissues, herein referred to as ‘testis, ovary or germline repressors’ (Fig. 1f). In contrast, the 769 motifs found from searches of control promoter sequences revealed 143 showing significant correlation with expression in the testis, ovary, or both tissues (Supplementary Fig. 1). However, these showed an even distribution between activators (75/143) and repressors (68/143) (two-sided exact binomial test vs 50%: *p* = 0.62), which is consistent with unbiased expression of the control genes used as input (Supplementary Fig. 1).

Results from the linear regression analysis highlighted that motifs found within the core promoter regions of germline genes (TC and OC) typically had the highest regression coefficients associated with gene activation in their respective tissues compared to those found in proximal regions (TP or OP) (Fig. 1c, d). Despite core promoter regions being approximately 1/3 the length of proximal promoter regions analysed, 30/59 of testis activators and 30/77 of ovary activators were respectively derived from core searches, whilst the remaining 29/59 of testis activators and 47/77 of ovary activators were from proximal searches (Fig. 1e). The biased representation of activator motifs in core promoter regions supports the concept that transcriptional regulation in the germline is strongly driven by core CREs close to the TSS^47,48^. On the other hand, proximal motifs (TP and OP) were also typically associated with activation in the corresponding germline but, interestingly, showed association with repression in the opposite germline tissue with 16/20 ovary repressors originating from TP, and the only testis repressor from OP (Fig. 1f). This observation suggests two hypotheses: i) that repressive gene regulation in the opposite sex may be primarily governed by CREs localised in proximal promoters, or ii) they are a signature of sex-specific germline gene evolution acting at these CREs^19,49,50^. Furthermore, putative activators and repressors did not show any overrepresentation in origin from specific or enriched motif searches (activators: 93 specific and 85 enriched, repressors: 12 specific and 16 enriched), indicating that both searches produce significant and potentially functional motifs (Fig. 1e, f).

### Putative germline CREs show biased distribution within promoter sequences of germline-enriched genes

**Fig. 2:**
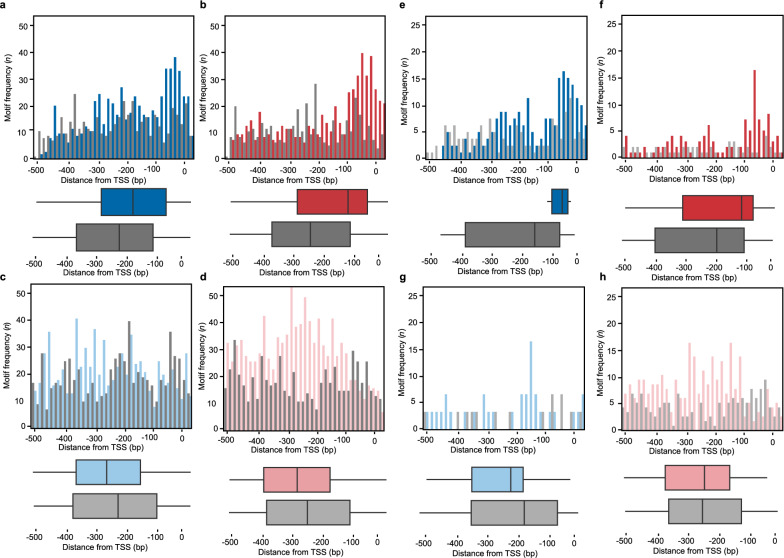
Midpoint position distribution of significant motifs in putative promoter regions of testis- and ovary-enriched genes. Distribution of significant (*p* ≤ 0.05) (**a**) TC, (**b**) OC, (**c**) TP, (**d**) OP motifs and highly significant (*p* ≤ 0.001) (**e**) TC, (**f**) OC, (**g**) TP, and (**h**) OP motifs within the putative promoters (−500 bp to +30 bp relative to TSS) of genes enriched in testis (blue bars) and ovary (red bars) and within the respective control genes (grey bars). Motif frequencies found from core promoter searches are shown as dark-shaded bars (**a**, **b** for significant core motifs; **e**, **f** for significant proximal motifs) and motif frequencies from searches in proximal promoter regions are represented by light-shaded bars (**c**, **d** for highly significant core motifs; **g**, **h** for highly significant proximal motifs). Box plots represent the 1^st^ interquartile to 3^rd^ interquartile ranges of the respective motifs distributions with the vertical line representing the median motif distribution, and are coloured following the same colour scheme.

We next examined the distribution of germline-derived motifs within their respective putative promoter regions (−500 bp to +30 bp relative to the TSS) of the testis- and ovary-enriched genes used in the motif searches, as well as within the equivalent region of control genes. Significant TC or OC motifs showed a biased clustering in proximity (−100 bp to +30 bp) to the TSS of the corresponding germline-enriched genes, but unbiased distribution in the control genes (Fig. 2a, b). Likewise, TP and OP motifs were predominantly distributed >150 bp upstream of the TSS of the corresponding germline-enriched genes and also showed unbiased distribution in control genes (Fig. 2c, d). The distinct core and proximal clustering were even more pronounced when only highly significant motifs (linear regression *p* ≤ 0.001) were used for the analysis (Fig. 2e–h). Collectively, these findings reinforce the necessity for precise spatial positioning of core and proximal CREs within the promoter sequence to ensure their effective regulatory function^51^.

### Copy number of CREs can influence gene expression in the germline

**Fig. 3:**
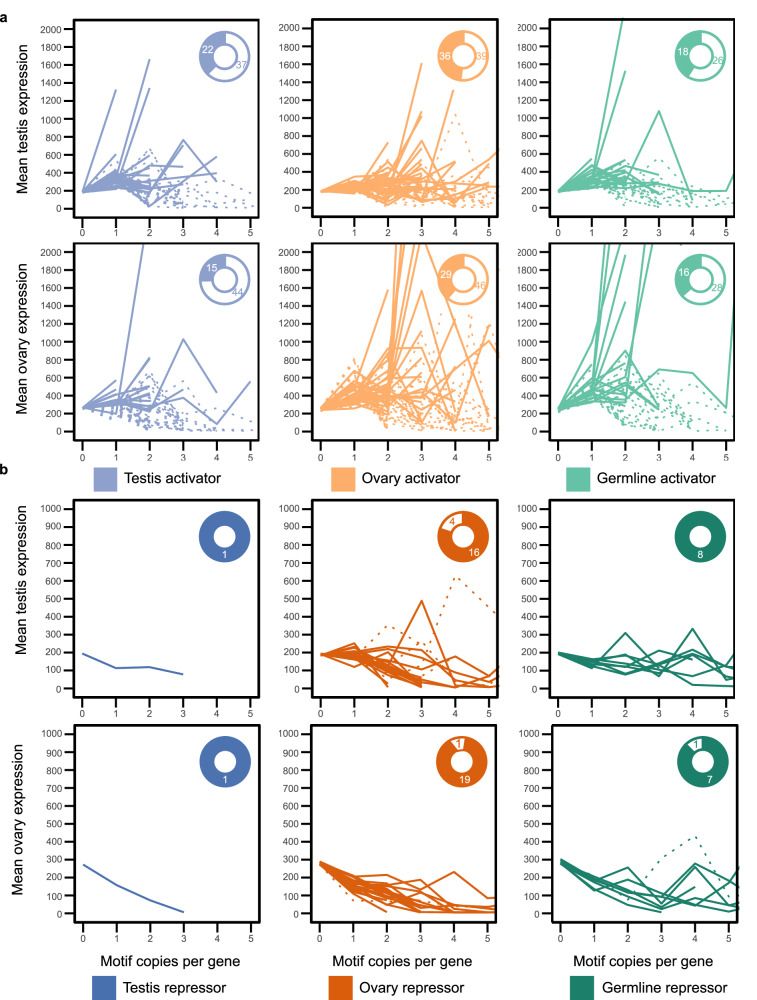
Correlation between motif copy number and gene expression in the germline. Line graphs summarising the relationship between the copy number of each significant motif (*p* ≤ 0.05) and the mean expression of genes respectively carrying 0, or 1+ copies of the motif for: (**a**) activator motifs and (**b**) repressor motifs and their correlation with testis expression (top) and ovary expression (bottom). Activator motifs with a positive correlation and repressor motifs with a negative correlation were defined using simple linear regression with no *p*-value threshold. Slope > 0 indicates positive correlation and slope ≤ 0 indicates no or negative correlation. For activator motifs solid line and solid fill on the pie chart indicate a positive correlation, and dotted line and no fill on the pie chart indicate no or negative correlation. For repressor motifs solid line and solid fill on the pie chart indicate no or negative correlation, and dotted line and no fill on the pie chart indicate a positive correlation. A copy number threshold at 5+ copies has been applied to the plots after analysis for visual purposes.

Previous studies have shown that CRE copy number within a promoter region can have either positive or negative effects on gene expression depending on their regulatory role. For example, this may occur through increased recruitment of *trans*-acting proteins that enhance transcription, or conversely through reduced transcriptional output caused by titration of these factors across multiple binding sites^52^. In the latter case, the presence of multiple sites causes competition for TF binding, thereby preventing optimal transcriptional activation, particularly if the TFs are present at limiting concentrations ^53^. Therefore, we investigated correlations between the presence of multiple copies of the same motif and the expression of genes carrying these in their promoter regions (Supplementary Data 3). For each significant motif identified, we independently quantified the mean expression of genes carrying 0 to 1+ copies in the 500 bp sequence upstream to the TSS. Our results evidence that over a third of the testis activators had a positive correlation between copy number and gene expression in the testis (22/59) and in the ovary (15/59), although the latter in a smaller proportion. A similar pattern was observed for ovary activators, where almost half of these showed a positive copy number correlation with expression in the ovary (36/75) and a slightly lower number correlating with testis expression (29/75). On the other hand, germline activators also showed similar results with 18/44 positively correlated to expression in the testis, and a similar number (16/44) positively correlated with ovary expression, suggesting multicopy CRE regulation being equally evolved and/or common in both sexes (Fig. 3a). Interestingly, a proportion of testis activator motifs with positive copy number correlation with testis expression (9/22) also displayed a negative correlation with expression in the ovary. A similar number of ovary activators (7/36) had a positive correlation with ovary expression as well as a negative correlation with expression in testis (Fig. 3a). In contrast, almost all the significant repressors found showed a negative correlation between copy number and gene expression in both germline tissues (24/29) as well as their corresponding tissues (1/1 testis repressors negatively correlated with testis expression, 19/20 of ovary repressors with ovary, 8/8 of germline repressors negatively correlated with testis expression whilst 7/8 negatively correlated with ovary expression). Overall, these results indicate a distinctive relationship between motif copy number and transcriptional activation or repression role. Motifs significantly correlated with gene activation rarely showed increased correlation with expression when 2 or more copies are present in the promoter (Fig. 3a), consistent with a single-copy regulation typical of TF binding sites. On the other hand, motifs significantly correlated with reduced expression generally maintain a linear relationship supporting a different regulation mechanism acting on *cis*-repressors (Fig. 3b). Furthermore, the repetitive nature of many repressor motifs (Figs. 1f, 3b), whilst possibly affecting the ability to realistically measure copy number variations, also suggest a role in epigenetic mechanisms of gene silencing and heterochromatin formation that are often associated with repetitive DNA elements^54,55^.

### Genome-wide distribution of CREs reveals chromosome enrichment patterns

**Fig. 4:**
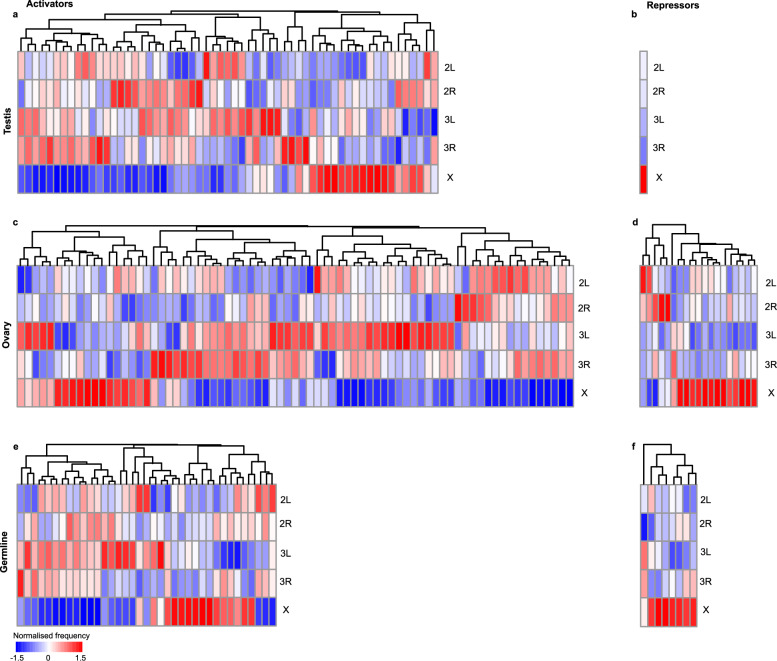
Chromosome distribution of promoter regions containing putative activator and repressor motifs significantly correlated with testis and/or ovary expression. Heatmap showing the normalised frequency of motif distribution on autosomal chromosome 2, 3 left (L) and right (R) arms and sex chromosome X for (**a**) testis activators, (**b**) testis repressors, (**c**) ovary activators, (**d**) ovary repressors, (**e**) germline activators, and (**f**) germline repressors. Motif frequency was normalised by the number of genes on each chromosome arm (2 L = 2041, 2 R = 2638, 3 L = 1324, 3 R = 1760, X = 852). Hierarchical clustering using Euclidean distance was applied to cluster motifs with similar chromosomal distributions.

To further investigate potential association with chromosome-specific mechanisms of gene regulation^11,12^, we analysed the genome-wide distribution of motifs showing significant correlation with germline expression (Fig. 1). Overall, activator motifs positively correlated with expression in testis, ovary or both germline tissues were broadly distributed on autosomes and the X chromosome with several motifs showing enrichment on specific chromosome arms (Fig. 4a, c, e and Supplementary Fig. 2). Interestingly, repressor motifs including the only testis-specific repressor identified, and most of the ovary-specific (15/19) and germline motifs (7/8) showed an increased enrichment on the X chromosome further supporting their potential role in sex chromosome specific regulation (Fig. 4b, d, f and Supplementary Fig. 2).

### Co-occurrence of CREs reflects distinct expression patterns during spermatogenesis

**Fig. 5:**
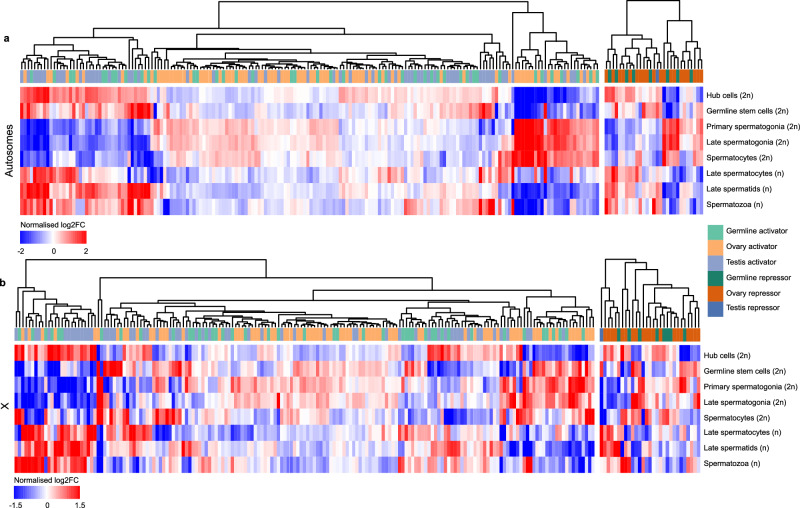
Differential expression analysis at distinct spermatogenic stages of genes carrying activator and repressor motifs. Clustered heatmap displaying differential expression (*z*-score of average log2 FC) of genes containing one or more copies of putative CREs significantly associated with repression or activation in testis and/or ovary tissues of (**a**) autosomal and (**b**) X-linked genes. Rows, and columns represent differential expression profiles for each motif. Coloured bars at the top of each heatmap indicate the corresponding motif category (testis, ovary or germline activator or repressor). Scaling has been applied to rows and Euclidean distance clustering has been applied to columns of each heatmap.

To gain further insight into motif co-occurrence and potential cell-type specific roles, we investigated the association between the presence of significant activator and repressor motifs with gene expression during spermatogenesis using the single-cell transcriptome we developed previously from *A. gambiae* testis^11^. We observed that testis activators as well as germline and ovary repressors were typically enriched in autosomal and X-linked genes with similar expression patterns, indicating their co-occurrence in the regulatory regions of these genes. Notably, these motifs—including the only testis repressor motif found—are primarily correlated with enriched pre- and/or post-meiotic expression as well as meiotic downregulation. This further corroborates a putative role in MSCI as suggested by our chromosome enrichment analysis, which also demonstrated an enrichment of most of these motifs on the X chromosome. Conversely, genes expressed during male meiosis showed enrichment for ovary and germline activator motifs on both autosomes and the X chromosome, which is indicative of shared or conserved mechanisms of gene regulation between the male and female germline (Fig. 5).

Moreover, we identified a distinct correlation between autosomal and X-linked genes and their expression in hub cells (HCs) and germline stem cells (GSCs). Motifs found in X-linked genes clustered in two distinct groups with differing expression patterns: i) testis activators and ovary repressors showing downregulated expression from GSCs onwards and postmeiotic expression in late spermatocytes (Fig. 5b, left), and ii) germline and ovary activators showing downregulated expression in HCs and late spermatocytes onwards, but increased expression from GSCs to spermatocytes (Fig. 5b, right). These findings suggest that these putative CREs may exert cell-type specific functions at distinct stages of spermatogenesis.

### De novo CRE mapping reflects expression profiles of characterised germline promoter regions

**Fig. 6:**
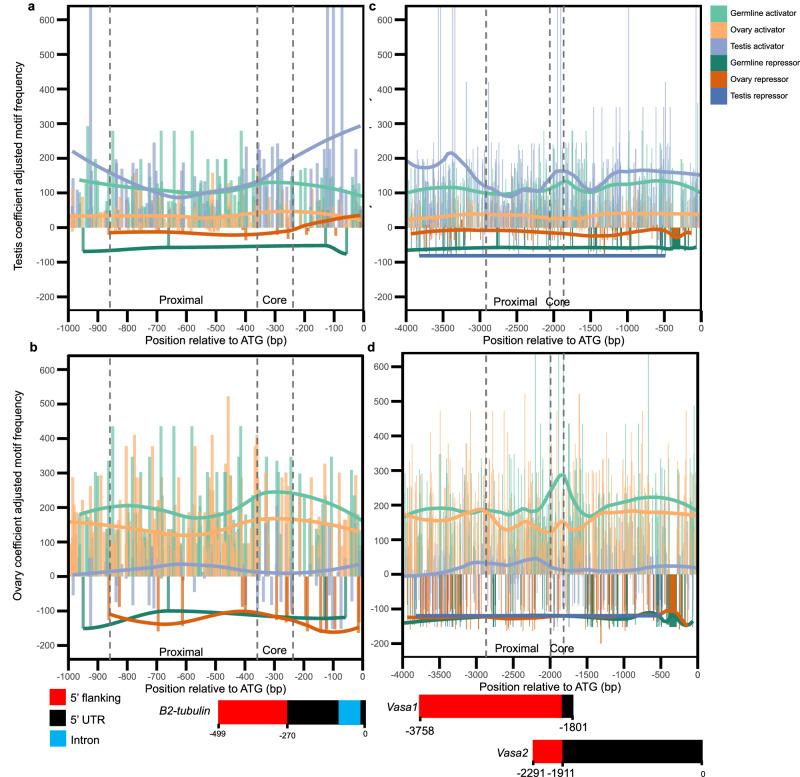
Motif frequency profiles in the 5’ flanking regions of the *β2-tubulin* and *vasa* genes. Motif frequencies in the −1000 bp region upstream of the *β2-tubulin* gene adjusted by (**a**) testis regression coefficients and (**b**) ovary regression coefficients, and the −4000 bp region upstream of the *vasa* gene adjusted by the (**c**) testis regression coefficients and (**d**) the ovary regression coefficients. Coloured bars and smoothing lines within the plots are indicative of the motif category based on the regression analysis (Fig. 1). Smoothing lines have been imposed using a loess smoothing method to highlight local trends for each motif category based on their frequency adjusted by testis (top graphs) or ovary (bottom graphs) regression coefficient. A threshold has been applied to the maximum adjusted motif frequency value that is displayed after smoothing and subsequent plotting for visual purposes. Putative core (−100 bp to +30 bp) and proximal (−500 bp to −101 bp) are indicated by vertical dotted lines. Horizontal bars below the plots represent the annotated regions of previously characterised *β2-tubulin*^17^ and *vasa*^9^ promoters, with TSS-proximal 5’ flanking regions indicated in red, 5’ UTR in black, and blue indicates the intron present within the *β2-tubulin* 5’ UTR.

Following the characterisation of their genome-wide distribution and the correlation with germline gene expression, we investigated the presence of significant germline motifs within defined promoter regions. Given that the mere presence of a motif does not necessarily imply functional significance, we quantified motif frequencies within previously characterised germline-specific promoters, following adjustment using testis and ovary regression coefficients (Fig. 1). We first applied this analysis to the 1000 bp sequence upstream of the male meiosis-specific *β2-tubulin* gene, which contains the shorter germline promoter sequence characterised to date in *A. gambiae* (499 bp including a 270 bp 5’ UTR)^17^, which has also been used to successfully express X-chromosome targeting endonucleases specifically during male meiosis^13–15^. We first searched the 1 kb sequence for all the significant activators and repressors (specific and enriched) which highlighted a striking correspondence between the characterised promoter sequences and the increased density of testis activators adjusted by testis regression coefficients (Fig. 6a, Supplementary Data 4). The pattern appears even more evident when only specific motifs are included in the analysis (Supplementary Fig. 3). In addition, the majority of the significant motifs originating from core promoter regions of testis specific genes searches (TC, Fig. 1) are mostly located in close proximity to the intron (−8 bp to –82 bp) within the 5’ UTR region (Fig. 6a, Supplementary Fig. 3), indicating the ability of our search to detect regulatory elements within UTR exons. A parallel motif search within the same sequence, but using the frequencies adjusted by ovary regression coefficients, confirmed a moderate concentration of germline activators and ovary repressors, as expected for a male germline-specific promoter such as *β2-tubulin* (Fig. 6b).

We further validated the promoter refinement approach using the 4000 bp sequence upstream of the start codon of the *vasa* gene, which contains the 2291 bp promoter region used to express homing gene drives in the germline of *A. gambiae* males and females (*vasa2*)^3,9^ and the 1957 bp sequence able to activate low levels of expression only in the male germline (*vasa1*)^9^. The motif search adjusted for testis regression coefficient evidenced an increased density of testis activators within the male germline-specific *vasa1* region, with the second peak being found within the 5’ UTR sequence also contained within the *vasa2* region (Fig. 6c), consistent with both promoters being expressed in the testis. In addition, analysis of ovary adjusted frequencies evidenced a predominant distribution of germline motifs with a peak overlapping the core promoter region that is fully captured within the *vasa2* sequence, which reflects the corresponding expression profile in both male and female germline (Fig. 6d). Altogether, these results further validate the utility of our approach for its applicability to the identification and refinement of functional tissue-specific promoter regions.

### In vivo characterisation validates the role of a putative testis activator at the nucleotide-level

**Fig. 7:**
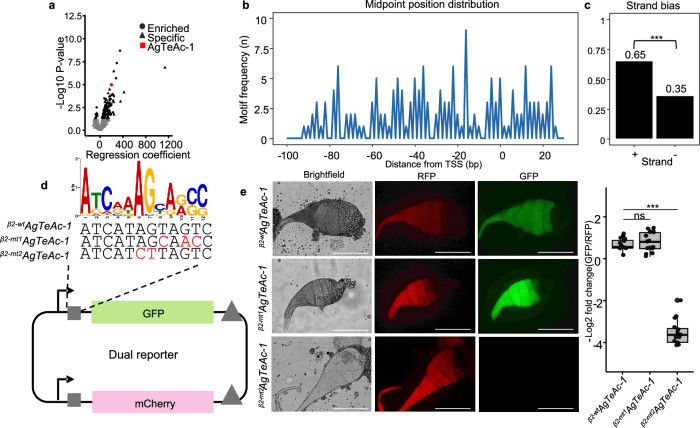
Functional characterisation of a putative CRE in the *β2-tubulin* promoter. **a** Volcano plots of regression analysis for testis expression, with grey circles showing insignificant motifs, black circles showing significant motifs and the red circle highlight being the *AgTeAc-1* motif. **b** Midpoint position distribution of the *AgTeAc-1* motif in core promoter regions (left) and (**c**) strand bias (right). A binomial test was conducted on the strand bias (*p* = 1.56 × 10⁻²⁰). **d** *AgTeAc-1* position weight matrix (top), the wild-type and mutant sequences (middle) used in the dual reporter (bottom) which contains these motif variations in the *β2-tubulin* promoter expressing EGFP, and the wild-type *β2-tubulin* promoter expression mCherry (RFP). The dual reporter also contains piggyBac repeats shown by grey triangles. **e** Fluorescence imaging of dissected mosquito testis from transgenic males carrying the following constructs: (top) ^*β2-wt*^*AgTeAc-1*, (middle) ^*β2-mt1*^*AgTeAc-1*, and (bottom) ^*β2-mt2*^*AgTeAc-1*. Red/green look up tables have been applied to the respective RFP and GFP images. The white scale bar represents 200 μm distance. Relative quantification between GFP and RFP fluorescence measured from whole dissected testis (*n* = 15). Box plots showing the GFP/RFP -log2 fold change values for each group, with median values indicated by horizontal lines, box spanning the interquartile range, whiskers showing 1.5x interquartile range, and dots representing data points from individual measurements for each testis analysed. Statistical analysis was performed between each mutant sample (^*b2-mt1*^*AgTeAc-1*, and ^*b2-mt2*^*AgTeAc-*1) and the wild-type (^*b2-wt*^*AgTeAc-1*) samples using a two-sample Student’s t-test, and significance is indicated above each comparison.

Finally, to functionally validate our approach in vivo, we selected one of the most significant testis activators—herein referred to as *AgTeAc-1*—that showed a biased location ~20 bp upstream of the TSS in promoter regions containing the motif and highly significant coding strand bias (Fig. 7a–c). In addition to a mild overrepresentation on X-linked genes and conservation across other insect species, gene ontology analysis showed a biased protein binding and protein coding functions among *AgTeAc-1* carrying genes (Supplementary Fig. 4). Considering its presence at −15 bp of the TSS in the *β2-tubulin* promoter used for testis-specific expression in *A. gambiae*, the *AgTeAc-1* represented an ideal candidate for in vivo characterisation.

We therefore used a dual-fluorescence-reporter assay to measure the effect of site-specific mutagenesis at the *AgTeAc-1* motif sequence (ATCATAGTAGTC) on the *β2-tubulin* promoter expression. To mitigate stochastic bias arising from genomic position effects and batch‑ or individual‑level variation in expression, we adopted a dual‑reporter design in which a wild‑type copy of the *β2‑tubulin* promoter driving mCherry (RFP) provides internal normalisation for EGFP (GFP) expressed from a wild‑type or CRE‑mutant *β2‑tubulin* promoter within the same construct. This configuration enables direct analysis of mixed transgenic progenies, which are expected to carry a diverse range of genomic insertions based on our previous experience using the same transposase-mediated integration system^13,14^, although this was not explicitly tested in the present study. The dual-reporter system we adopted therefore provides an efficient means to assess multiple integration events simultaneously, without the need to isolate and characterise each insertion individually.

We generated three constructs all containing a wild-type copy of the RFP-driving *β2-tubulin* promoter and GFP under the transcriptional control of either: i) a wild-type copy of the same promoter (^*β2-wt*^*AgTeAc-1*), ii) a mutant copy of the promoter carrying 3 nt changes at lowly conserved nucleotides of the motif (^*β2-mt1*^*AgTeAc-1*), or iii) a mutant with a 2 nt base pair change at highly conserved nucleotides of the same motif (^*β2-mt2*^*AgTeAc-1*) based on the corresponding motif position weight matrix (PWM) (Fig. 7d). Each plasmid construct also contains piggyBac inverted terminal repeats for transposase-guided genomic insertion as well as a DsRed marker under the 3xP3 promoter (active in insect eyes and nervous system) to ease identification of transgenic insects. We generated *A. gambiae* transgenic lines carrying independent genomic insertions of the three dual-reporter constructs which allowed us to measure and normalise GFP/RFP expression in their corresponding dissected testes (Supplementary Data 5). Transgenic testes carrying the lowly conserved mutant ^*β2-mt1*^*AgTeAc-1* show no significant effect on EGFP expression when compared to wild type. However, transgenic testes carrying the highly conserved mutant ^*β2-mt2*^*AgTeAc-1* show a drastic reduction in EGFP expression consistent with these nucleotides within the *AgTeAc-1* motif being key for *β2-tubulin* expression, as predicted by  the PWM (Fig. 7e). To exclude the possibility that the absence of EGFP expression could have resulted from non-canonical piggyBac-mediated transposition events—reported in other mosquito species^56,57^ but, to the best of our knowledge, not detected in *A. gambiae*^13,14^—we confirmed the presence of the dual-reporter genomic insertion and the sequence integrity of the *β2-tubulin::EGFP* cassette in individual mosquitoes. These individuals were simultaneously analysed for fluorescence expression in dissected testes (Supplementary Fig. 5, Supplementary Fig. 6), further supporting that the reduction in EGFP expression was due to the mutations introduced within the *AgTeAc-1* motif. Together, these results emphasise the ability of our method to identify functional motifs associated with tissue-specific expression and to predict the contribution of the individual nucleotides within them.

## Discussion

The ability to identify tissue- and/or cell-type-specific regulatory elements is key to the development of safe and effective gene editing tools, including those aimed at controlling harmful insects. In *Anopheles* mosquitoes, CRE motif discovery has largely depended on comparative analyses^40^ and standard chromatin-accessibility pipelines^41–45^, with limited direct application to germline tissues. Although informative, these methods remain difficult to apply in non-model insects due to sparse genome annotations, limited sequence resolution, and the lack of robust in vivo validation tools. To aid filling this crucial gap, we developed an in silico approach to identify and functionally characterise CRE motifs in predefined gene sets, sharing tissue-specific gene expression patterns, by integrating motif discovery and their correlation with gene expression.

We applied this method to the germline of the malaria mosquito *A. gambiae*, where spatiotemporal control of germline gene expression is essential for engineering genetic control technologies. This allowed the identification of hundreds of short DNA motifs within core and proximal promoter regions that were associated with germline expression in the testis, ovary or both tissues. Motif distribution analysis revealed that core promoters were enriched with activator motifs strongly associated with increased gene expression in the testis and/or ovary. This finding aligns with the general knowledge of CREs located in close proximity to the TSS playing a critical role in transcriptional activation, canonically functioning as TF binding sites, as previously described in *Drosophila*^51,58^ and other eukaryotes^47,59,60^. In addition, we found that proximal promoter regions are mostly enriched with activator motifs, with weaker correlation with gene expression, as well as motifs inversely correlated with germline expression, which we define as repressors. Interestingly, repressors were obtained from searches within the promoter regions of genes expressed in the germline of the opposite sex. These motifs may be regulated by sex-specific *trans*-acting repressors, directly or indirectly interacting with these sequences^61,62^, such as the *doublesex* (*dsx*) TF, which activates *Bric-à-brac* (*Bbab*) gene expression in males but represses it in females in the *Drosophila* soma^63^. Alternatively, these motifs could result from loss-of-function mutations creating spurious/non-functional motifs that no longer have the regulatory syntax required for expression^52,64^, which may represent evolutionary remnants of genes that were once expressed in both germlines but have since become sex-specific or biased^49^. This process has been described in insects^65^ including *A. gambiae* and generally referred to as X chromosome demasculinisation, an evolutionary process leading to the underrepresentation of male-specific or biased genes that become inactive or translocated from the X chromosome over time, which ultimately led to the evolution of sex chromosomes and sex-biased gene expression^19^. Our additional analysis of chromosomal distributions and co-occurrence found most repressor motifs enriched on the *A. gambiae* X chromosome and, in some cases associated with activators. These observations suggest a plausible involvement in sex-chromosome-specific regulatory pathways, such as meiotic silencing of unsynapsed chromatin (MSUC)^66,67^ and MSCI^11,12,18^. Some repressor motifs could serve as targets of epigenetic silencing, whereas certain activators may contribute to the enhanced expression of X-linked genes, for example, through premeiotic dosage‑compensation mechanisms that counterbalance subsequent meiotic repression^11,12,43,44^. Together, these results provide a first‑of‑its‑kind classification of putative germline CREs in the mosquito germline and offer early indications of how they may cooperate in regulating gene expression. Definitive mechanistic interpretation of the functions of individual motifs will nonetheless require further investigation and targeted functional validation.

In addition, we found that almost half of the activator motifs found were associated with increased expression when present in multiple copies in their respective tissues, which may indicate signatures of homotypic sites, where multiple TFBS enhance transcription through cooperative (e.g. half-sites) or additive binding^68,69^. In contrast, the vast majority of the repressor motifs were associated with decreased expression when present in multiple copies. This may be consistent with active multimeric recruitment of *trans*-acting repressors or, given the repetitive nature of some repressor motifs, indicate a role in chromatin-mediated repression^70^, e.g. non-canonical DNA methylation. A small proportion of the repressors showed no clear association between copy number and gene expression, further indicating that these may represent spurious TFBS evolved either following loss-of-function mutations as discussed above, or characterised by strict positional requirements for function^52,64^ also evidenced by the defined clustering of core CREs (Fig. 2).

A crucial objective of our study was to provide evidence of functional relevance of the putative CREs identified and their usefulness to determine and or refine specific promoter regions. Our analysis showed a clear overlap between motif presence and known expression patterns for the only two *A. gambiae* germline promoters which have been partially refined: *β2-tubulin*^17^ and *vasa*^9^. These insights could be applied to refine regions of new gene-drive candidate promoters using a similar approach, thereby helping to avoid  the inclusion of unintended CREs that may cause leaky expression when large intergenic regions are used^6,24^. Importantly, this approach will aid transfer of promoters to other species by enabling mapping of conserved motif positions or the identification of motifs through de novo searches. This in turn supports the refinement of promoters in mosquitoes and, by extension, provides a framework for promoter tuning in other eukaryotes. Notably, the method was validated using a relatively small input gene set, underscoring  its transferability to species for which limited genomic or expression data are available.

Finally, as proof of concept of the ability of our approach to predict nucleotide-level functionality, we tested one of the top testis activator candidates (*AgTeAc-1*) present in the core promoter region of the *β2-tubulin* promoter. Mutation of highly conserved nucleotides led to loss of transgene expression, whereas mutation of lowly conserved nucleotides had negligible effect. These results confirm that our method can successfully identify functional CREs and can also pinpoint essential or dispensable nucleotides within their sequence. Furthermore, we demonstrate that promoter activity can be modulated through precise nucleotide-level alterations, offering new methods to enhance or suppress expression of endogenous gene or transgene activity. In the context of genetic control, we also provide novel targets within regulatory regions of germline genes that could be used to disrupt germline formation hence applicable to the development of sterile insect techniques (SITs).

We also describe a practical dual‑reporter assay that enables more rapid screening of CREs directly in mixed transgenic individuals, without the need to isolate independent insertions, providing substantial savings in both time and resources. Nonetheless, while fluorescent markers offer a practical analytical solution, their use is limited to promoters capable of driving sufficiently strong reporter expression, thereby requiring prior validation of promoter strength—a resource that remains very limited in *A. gambiae*. This work could be further expanded through higher-throughput functional characterisation using massively parallel reporter assays (MPRAs), which are not yet available for mosquito germline studies. When combined with 3D genome (Hi-C) and chromatin accessibility (ATAC-seq) data^41,42^, such approaches would enable the extension of this framework to additional regulatory regions, including UTRs and enhancers, and ultimately support a more complete reconstruction of CRE function and spatiotemporal activity in the malaria mosquito germline.

In conclusion, our study provides a substantial foundation for improving the characterisation and manipulation of germline promoters in *A. gambia*e, with potential extension to other tissues and species. This will be highly valuable for informing promoter design in next-generation genetic tools for insect control. In principle, any biological system in which a subset of genes shares regulatory logic—and therefore shows enrichment for specific CREs linked to cell or tissue specificity, developmental timing, or function—could be amenable to this analysis. As with all CRE discovery approaches, in vivo functional validation remains essential to assess predictive accuracy across tissues, cell types, and species. The putative CREs and downstream analysis presented here support the guided selection of endogenous regulatory elements with reduced leaky expression relative to current gene-drive promoters. Additionally, these data may also inform the complex CRE syntax required for synthetic promoter design, enabling precise and fine-tuned regulatory activity in defined cell types and tissues. Although demonstrated here in malaria mosquitoes, this framework provides a basis for promoter engineering in other systems where achieving precise, cell-type specific promoters remains a major barrier.

## Methods

### Tissue-specificity index calculation

Microarray gene expression data were retrieved from MozAtlas^46^, and gene IDs were updated using the Ensembl Metazoa ID history converter (release 59)^71^ to match genes to the AgamP4 *A. gambiae sensu stricto* reference genome assembly^72^. The tissue specificity index (τ-value)^73^ for each gene was defined using expression data from MozAtlas^46^ as described by the equation below^73^. Genes with τ ≥ 0.8 in male tissues and with maximal expression in the testis were referred to as ‘testis-enriched’, and genes with τ ≥ 0.8 in female tissues and with maximal expression in the ovary as ‘ovary-enriched’. Genes with τ ≤ 0.2 from the analysis of male and female tissues and not showing maximal expression in the respective germline tissue were respectively referred as ‘controls’.

$$\tau =\frac{{\sum }_{i=1}^{N}\left(1-{x}_{i}\right)}{N-1};{x}_{i}=1-\frac{{x}_{i}}{\max \left(x\right)}$$

with *N* representing the number of tissues, and *x*_*i*_ represents the expression profile component normalised by the maximal component value.

### Promoter sequence retrieval

For all genes with annotated 5’ UTR as per current AgamP4 annotation (VectorBase release 68)^72^, regions spanning −100 bp to +30 bp from the TSS were retrieved and designated as putative ‘core promoters’. Regions spanning −500 bp to −101 bp upstream of the TSS were retrieved and designated as putative ‘proximal promoters’.

### Input gene selection

The top 100 testis- or ovary-enriched genes with annotated 5’ UTRs and highest *τ*-values were respectively selected for testis and ovary motif discovery and used as control sets for female and male control motif discovery. The bottom 100 control genes with annotated 5’ UTRs and lowest *τ*-values were selected as the control set for ‘specific’ motif discovery within the germline of the corresponding sex as well as for female and male control motif discovery. The putative core and proximal promoter regions for each gene group were used as motif discovery input sequences.

### Motif discovery

The STREME (Sensitive, Thorough, Rapid, Enriched Motif Elicitation) tool v5.5.4^74^ was used to perform motif searches. STREME was run with a motif width of 5–15 bp, the default Markov order determined from background nucleotide frequencies with search limited to 100 motifs. For each input sequence, two STREME searches were conducted: one using randomised background sequences generated from the input sequences (referred to as ‘enriched’ motifs), and another using the equivalent sequences from the respective control gene set (referred to as ‘specific’ motifs).

### Linear regression and motif distribution analysis

FIMO (Find Individual Motif Occurrences) v5.5.4^75^ on the MEME suite was used to map occurrences of motifs across the putative core and proximal regions. Motif occurrences were queried with a *p*-value threshold of *p* < 1 × 10^−4^, allowing for matches on both DNA strands.

For each motif, a binary matrix was generated to indicate presence or absence within the putative core and proximal promoter regions of all genes with an annotated 5’ UTR. A standard linear regression model was applied to the matrices for each motif using gene expression data for testis and ovary from the MozAtlas dataset^46^.

$${Y}_{i}=\beta 0+\beta 1\cdot {x}_{i}+\epsilon$$

where *Y*_*i*_ represents the gene expression level in the tissue, *β*0 is the gene expression level when the motif is absent, *β*_1_ is the regression coefficient when that motif is present, *χ*_*i*_ represents whether a motif is present or not, and *ϵ* is the random error component.

Motifs with regression *p* ≤ 0.05 and a positive regression coefficient in each respective tissue were categorised as putative ‘activators’, and those with *p *≤ 0.05 and a negative regression coefficient were categorised as putative ‘repressors’. Motifs with regression *p* ≤ 0.001 were designated as highly significant motifs. Proportions of activators and repressors were statistically compared using a two-sided binomial test vs. 50%.

FIMO (v5.5.4) was used to map the midpoint occurrences of the putative activator and repressor motifs across the entire putative core or proximal regions of the testis- and ovary-enriched genes used as input, and the corresponding control sequences.

### Chromosomal distribution analysis

Genes within the binary matrices were split by chromosomes. Genes mapping to the Y or unknown chromosome locations were excluded due to poor annotation. Genes with motifs in their respective promoter regions were counted for each chromosome and normalised by the total number of genes in the binary matrix for the corresponding chromosome. The resulting data were scaled by column, and hierarchical clustering with Euclidean distance was applied using the pheatmap package^76^ on RStudio Version 2024.12.1 + 563.

### Single-cell enrichment analysis

Log_2_ fold-change (log_2_FC) values for each cell cluster were retrieved from the *A. gambiae* testis single-cell RNA-sequencing dataset^11^. For each motif, a *z*-score of the average log_2_FC for each cell cluster was calculated for genes containing one or more copies of the motif based on the binary matrices. Data were scaled by row (*z*-score normalisation) and hierarchical clustering (Euclidean distance) was applied to motifs using the pheatmap package^76^ on RStudio Version 2024.12.1 + 563.

### Adjusted motif frequency profiling of individual promoter regions

FIMO queries were conducted applying a *p* < 0.001 significance threshold and allowing for location on both DNA strands, to map occurrences of significant activator and repressor motifs in the −1 kb region upstream the start codon of the *β2-tubulin* (AGAP008622) gene and −4 kb region upstream the start codon of the *vasa* (AGAP009578) gene. Each motif occurrence was adjusted by multiplying the frequency by the regression coefficient in the testis and ovary. Local trends in correlation with gene expression was visualised using loess smoothing on geomsmooth within the ggplot package on RStudio Version 2024.12.1 + 563.

### Plasmid construction

Plasmid components were amplified using primers containing Golden Gate overhangs (Supplementary Table 1). Specifically, the following targets and template were used: 1) the −499 bp region upstream of the *β2-tubulin* start codon was amplified from 50 ng of genomic DNA of G3 strain wild-type mosquitoes extracted using the Wizard Genomic DNA purification kit (Promega); 2) EGFP was amplified from 5 ng of a pre-existing plasmid and 3) SV40 terminator was amplified using 5 ng of a pre-existing plasmid with the 5’ end of the forward primer modified to contain a 10 nt sequence matching the endogenous *β2-tubulin* 3’ UTR. The fragments were assembled within a vector containing a piggyBac 3xP3::DsRed cassette, yielding construct pEC6 (*β2-tubulin::EGFP::SV40* and *3xP3::DsRed)*.

To generate mutant versions of the *β2-tubulin* promoter 5 ng of pEC6 plasmid was used as template, target sequences were amplified with primers as described in Supplementary Table 1. Then pEC6 and the amplified fragments were digested with *NdeI* and *HindIII* and ligated using T4 DNA ligase.

For each plasmid, including pEC6 and both mutants, we generated a dual reporter by adding in a *β2-tubulin::mCherry* cassette. This was amplified from 5 ng of pre-existing plasmid using primers listed in Supplementary Table 1. The amplified fragments (Supplementary Table 2), pEC6 and the mutant plasmids were digested with *XagI* followed by alkaline phosphatase treatment and T4 DNA ligation to generate the corresponding dual-reporter constructs: pEC48 (wild-type *β2-tubulin::EGFP* and wild-type *β2-tubulin::mCherry*), pEC58 (mutant 2 *β2-tubulin::EGFP* and wild-type *β2-tubulin::mCherry*), and pEC59 (mutant 1 *β2-tubulin::EGFP* and wild-type *β2-tubulin::mCherry*).

### Generation of transgenic lines

Approximately 400 *Anopheles gambiae* (G3 strain, herein referred to as wild type) embryos were injected with a solution containing 20 ng/μL of the reporter construct and 200 ng/μL of a *vasa2*::transposase helper plasmid. F_0_ larvae showing transient 3xP3::DsRed expression were selected, and surviving adults were crossed to wild type, whilst negative F_0_ adults were crossed together. The F_1_ progenies were screened for 3xP3::DsRed expression, confirming successful integration of the donor plasmid. Maintenance of each line was conducted by crossing females or males from each generation to wild-type males or females, respectively. Confirmation of the integrity of the GFP cassette inserted in the genome and the presence of the respective mutated motif was performed for two individuals for ^*β2-wt*^*AgTeAc-1* and ^*β2-mt1*^*AgTeAc-1*, and three individuals for ^*β2-mt2*^*AgTeAc-1* via PCR and Sanger sequencing using the primers listed in Supplementary Table 3, in parallel with GFP and RFP imaging of the testes from the corresponding individuals.

### Fluorescence imaging and quantification

Testes from heterozygous F_3_ pupae were dissected in PBS, mounted under cover slips, and imaged using an EVOS FL microscope using 20x magnification, 250 ms exposure and 10% gain on GFP (470 nm/525 nm), RFP (531 nm/593 nm) and brightfield filters (50%). Fifteen testes were analysed per transgenic line using Fiji^77^ v2.16.0 by selecting the perimeter from individual testis using the brightfield image and measuring fluorescent intensity values of each 8-bit TIFF image for RFP and GFP within the full testis area, respectively. The GFP/RFP fluorescence was calculated as -log2(GFP/RFP) for each testis. Statistical analysis was performed using a two-tailed Student’s t-test comparing each mutant line to the wild-type using RStudio 2024.12.1 + 563.

### Statistics and Reproducibility

Standard linear regression models were applied independently to each motif, with subsequent regression coefficients and *p*-values obtained as described above. Motifs were classified based on the regression significance thresholds (*p* ≤ 0.05) with the direction of the regression coefficient determining the categorisation as putative activators or repressors. Differences in the proportions of activator and repressor motifs were evaluated using a two-sided binomial test with a null hypothesis of 50%.

Motif discovery and occurrence analyses were performed using STREME and FIMO within the MEME suite web platform, using default statistical settings unless stated otherwise. For clustering analyses in the chromosomal distribution and single-cell enrichment, data were z-score normalised and hierarchical clustering performed using Euclidean distance. Heatmaps were generated using the pheatmap^76^ package under default settings.

For in vivo functional assays, all mosquitoes were reared under identical conditions and subsequent imaging performed on the same microscope using the same settings (20x magnification, 250 ms exposure, 10% gain). For each line, fifteen testes (n = 15) were analysed from individual pupae, with each testis treated as an independent biological replicate. Comparisons between the quantifications from each line were performed using unpaired, two-tailed Student’s t-tests.

All genome-based analyses, including gene information such as 5’ UTR annotation and promoter length were performed using publicly available resources as detailed above. Parameters, thresholds, and software versions of these web platforms are reported above where applicable to ensure full reproducibility of the computational components of this work.

### Reporting summary

Further information on research design is available in the Nature Portfolio Reporting Summary linked to this article.

## Supplementary information

Supplementary Information

Description of Additional Supplementary Files

Supplementary Data 1

Supplementary Data 2

Supplementary Data 3

Supplementary Data 4

Supplementary Data 5

Reporting Summary

Transparent Peer Review file

## Data Availability

All analyses conducted here can be performed using the Supplementary Data provided alongside the listed datasets below using the tools described in this paper. Supplementary Data 1 and Supplementary Data 2 are the source data for Fig. 1 and Fig. 2; Supplementary Data 1 and Supplementary Data 3 are the source data for Fig. 3, Fig. 4 and Fig. 5; Supplementary Data 4 are the source data for Fig. 6; and Supplementary Data 5 is the source data for Fig. 7. The microarray data for this paper is available online from the following sources either from VectorBase^72^, the original paper^46^ or NCBI under the accession number GSE21689. The single-cell data used to perform downstream analyses is available from the original paper^11^ or NCBI under the accession number PRJNA971569.
