# Supplementary material for: Decoding cis-regulatory elements in the germline of the human malaria vector Anopheles gambiae
Source: Commun Biol. 2026 May 2;9:917. doi: 10.1038/s42003-026-10117-y (PMC13342555; doi:10.1038/s42003-026-10117-y)

Supplementary Information

Supplementary Table 1: Primers used to construct plasmids.

wtB2-pr-F	caccacaacctgcgactcattctagcgttcataattgatatagt
wtB2-pr-R	caccacaacctgcgactctcgagcttgatatctttcgaaactgtg
EGFP-F	caccacaacctgcgactcgagatggtgagcaagggcgag
EGFP-R	caccacaacctgcgactttaattaattacttgtacagctcgtcca
SV40-F	caccacaacctgcgactttaactaaagctaagactctagatcataatcagcc
SV40-R	caccacaacctgcgactgagaaagatacattgatgagtttggaca
$^{\text{B2-mt}}$(AgTeAC-1)-F	cgaaatcgataagcttggatcc
$^{\text{B2-mt1}}$(AgTeAC-1)-R	gcacgtacggctcatatggttgctatgatcatcttttgctggttcggc
$^{\text{B2-mt2}}$(AgTeAC-1)--R	gcacgtacggctcatatgactaagatgatcatcttttgctggttcggc
B2-mCherry-F	cttcctactgcaggtctagaactagtggatcccc
B2-mCherry-R	tcttcctgcagtaggatgggtgaggtggagtac

Supplementary Table 2: B2-tubulin, EGFP, and terminator sequences within constructs.

Wt B2-tubulin promoter	ctagcgttcataattgatatagttttgtaaatgacatgacagttttttattttttttttatccataattacgaattgaacaactct acacacatattaattgcaagaacttatgctacataatatggaggaaagtggatgcatcatcccatccaagaagacatac gaatttatttgtggcatcgcaatcggccgaaccagcaaaagatgATCATAGTAGTCatatgagccgtacgtgccgga tcatttcgtgcagaaccttcagagacgttggtcgacagattgatagaaactgtgtagttagtccattcacaaagttgttcca ttagggacaaaagaaaaaaacggcttaaactagaaatttgtgtacccagtaggaatcgcgtattcggccgatagaggtc cttccgtaagtattcccagcgcttagagagcaacgctcgtgcgttccaaaatccgctaaatatcaaacggctttcacag tttcgaaagatatcaag
Mut 1 B2-tubulin promoter	Ctagcgttcataattgatatagttttgtaaatgacatgacagttttttattttttttttatccataattacgaattgaacaactct Acacacatattaattgcaagaacttatgctacataatatggaggaaagtggatgcatcatcccatccaagaagacatac gaatttatttgtggcatcgcaatcggccgaaccagcaaaagatgATCATAGCAACCatatgagccgtacgtgccgg atcatttcgtgcagaaccttcagagacgttggtcgacagattgatagaaactgtgtagttagtccattcacaaagttgttc cattagggacaaaagaaaaaaacggcttaaactagaaatttgtgtacccagtaggaatcgcgtattcggccgatagag gtccttccgtaagtattcccagcgcttagagagcaacgctcgtgcgttccaaaatccgctaaatatcaaacggctttca cagtttcgaaagatatcaag
Mut 2 B2-tubulin promoter	Ctagcgttcataattgatatagttttgtaaatgacatgacagttttttattttttttttatccataattacgaattgaacaactct Acacacatattaattgcaagaacttatgctacataatatggaggaaagtggatgcatcatcccatccaagaagacatac gaatttatttgtggcatcgcaatcggccgaaccagcaaaagatgATCATCTTAGTCatatgagccgtacgtgccggat catttcgtgcagaaccttcagagacgttggtcgacagattgatagaaactgtgtagttagtccattcacaaagttgttccat tagggacaaaagaaaaaaacggcttaaactagaaatttgtgtacccagtaggaatcgcgtattcggccgatagaggtcc ttccgtaagtattcccagcgcttagagagcaacgctcgtgcgttccaaaatccgctaaatatcaaacggctttcacagttt

	cgaaagatatcaag
EGFP	atggtgagcaagggcgaggagctgttcaccggggtggtgcccatcctggtcgagctggacggcgacgtaaacggccac<br>aagttcagcgtgtccggcgagggcgagggcgatgccacctacggcaagctgaccctgaagttcatctgcaccaccggc<br>aagctgcccgtgccctggcccaccctcgtgaccaccctgacctacggcgtgcagtgcttcagccgctaccccgacca<br>catgaagcagcacgacttcttcaagtccgccatgcccgaaggctacgtccaggagcgcaccatcttcttcaaggacga<br>cggcaactacaagacccgcgccgaggtgaagttcgagggcgacaccctggtgaaccgcatcgagctgaagggcatc<br>gacttcaaggaggacggcaacatcctggggcacaagctggagtacaactacaacagccacaacgtctatatcatggc<br>cgacaagcagaagaacggcatcaaggtgaacttcaagatccgccacaacatcgaggacggcagcgtgcagctcgcc<br>gaccactaccagcagaacacccccatcggcgacggccccgtgctgctgcccgacaaccactacctgagcacccag<br>tccgccctgagcaaagaccccaacgagaagcgcgatcacatggtcctgctggagttcgtgaccgccgccgggatcact<br>ctcggcatggacgagctgtacaagtaa
SV40	gactctagatcataatcagccataccacatttgtagaggttttacttgctttaaaaaacctcccacacctccccctgaac<br>ctgaaacataaaatgaatgcaattgttgttgttaacttgtttattgcagcttataatggttacaaataaagcaatagcatcac<br>aaatttcacaaataaagcatttttttcactgcattctagttgtggtttgtccaaactcatcaatgtatctt
B2-tubulin terminator	Ctaaagctaaattgaacaccctaaattatgtgtaaaatttctgctaagcagcggtgttggggtcaataaaaatgtttttttcc<br>Actctattcgcttcgtttttgttgccatttctcagtttttgcttcgtactcatgtgtaaggattagtgcagtgatgggaagtagct<br>Ccgaagttttctggaatcgtttccggatagtaggttcggtattagtttccggaatcggctccggaattggttccggaattgatt<br>Ccgggatcagaattggctcaaaattctcatggagattcccagagtgatttcgcttctgaaacttcgtatttaattcaagaatt<br>Gatccccattctggagctaattccaattctggagtcaattctgattctgttacccgaacaaattgcgattcccaggtcaatg<br>cccgttccggggtngattctgattccggagttggaatcagctccggaattggaatcggtccttaaatcg

Supplementary Table 3: Primers used to confirm presence of cassette from genomic DNA via PCR and Sanger sequencing.

PCR-F	ctcatggagattcccagagtg
PCR-R	gacgcatgattatcttttacgtgac
Sequencing-F	cgaaatcgataagcttggatcc
Sequencing-R	cattgttggtcaacttcaaagtcc

Supplementary Fig. 1: Identification of control motifs correlated with gene activation and/or repression in the *A. gambiae* testis and ovary

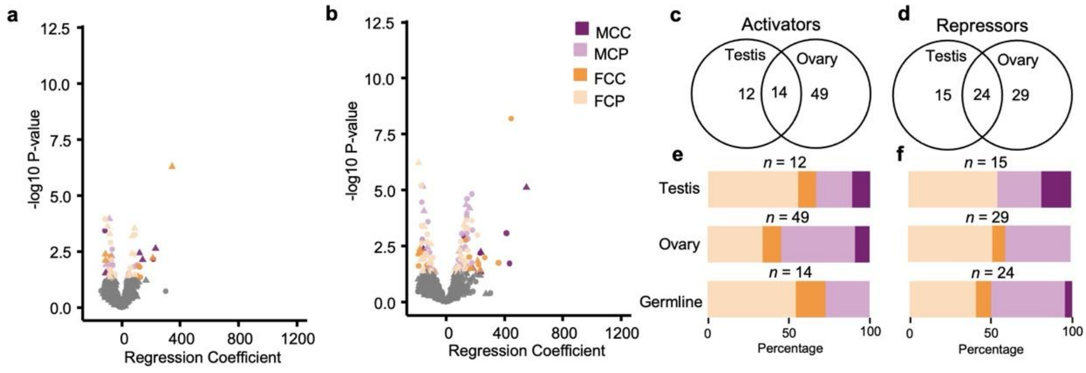

Supplementary Fig. 1: Volcano plots of linear regression analysis correlating gene expression in the testis and ovary with presence of motifs originating from: male control (MCC, MCP) and female control (FCC, FCP) gene sets and **a** testis expression and **b** ovary expression. For all volcano plots enriched motifs (circle) refer to those that originate from searches with no control sequences, and 'specific' motifs (triangle) refer to those with set control sequences. Coloured points represent motifs with linear regression $P \leq 0.05$ in the respective tissue, whilst grey points are motifs that did not meet this threshold. Venn diagrams of putative control derived **c** 'activator' motifs showing a positive regression coefficient in the respective testis and/or ovary analysis and d repressor motifs defined by a negative regression coefficient in the respective testis and/or ovary analysis. Stacked bar plots summarising the origin (MCC, MCP, FCC, and FCP) of each set of control motifs with **e** positive regression coefficients and **f** negative regression coefficients.

Supplementary Fig. 2: All motifs presented in chromosomal enrichment heatmap, split between activator and repressor motifs.

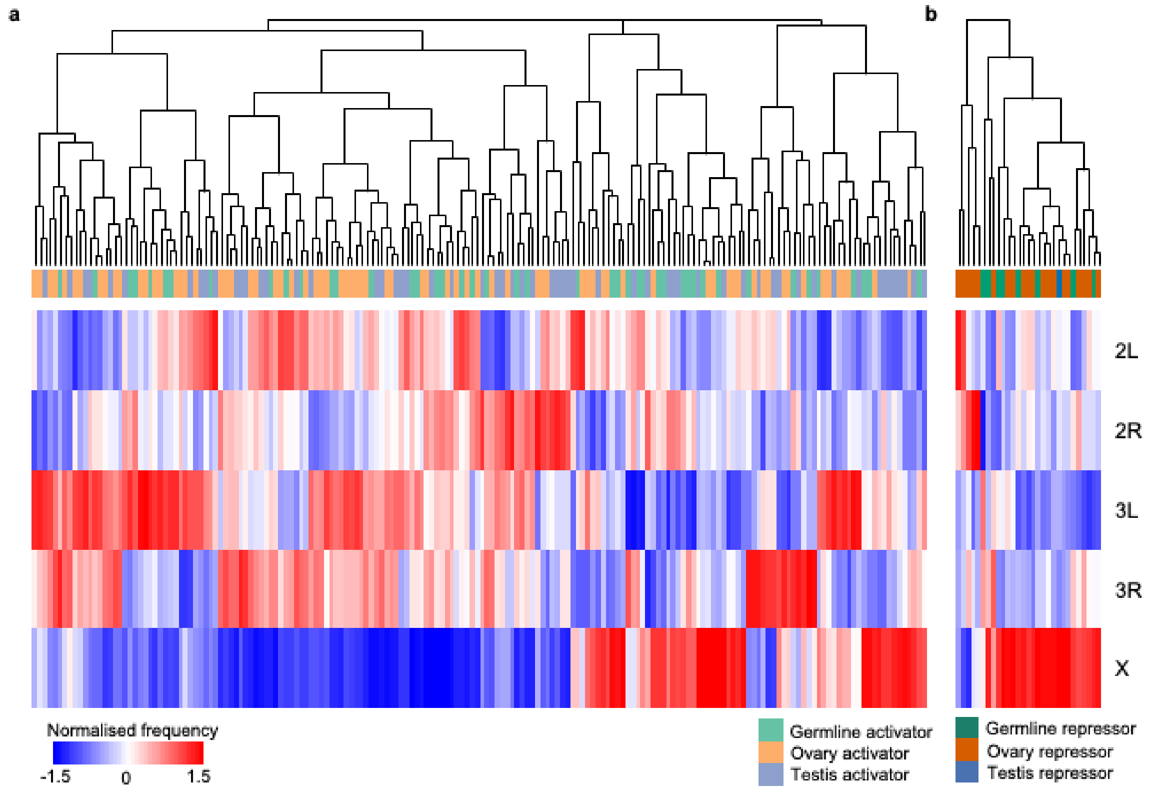

Heatmap showing chromosome enrichment of motifs significantly associated with gene **a** activation and **b** repression. Motif occurrence was normalised by the number of genes per chromosomes of those with annotated 5' UTR. Hierarchical clustering using Euclidean distance was applied to motifs and is shown as column clusters with similar chromosomal distributions.

Supplementary Fig 3: Motif frequency profiles in the 5' flanking regions of the *β2-tubulin* and *vasa* gene by motif origin, split by enriched and specific motifs.

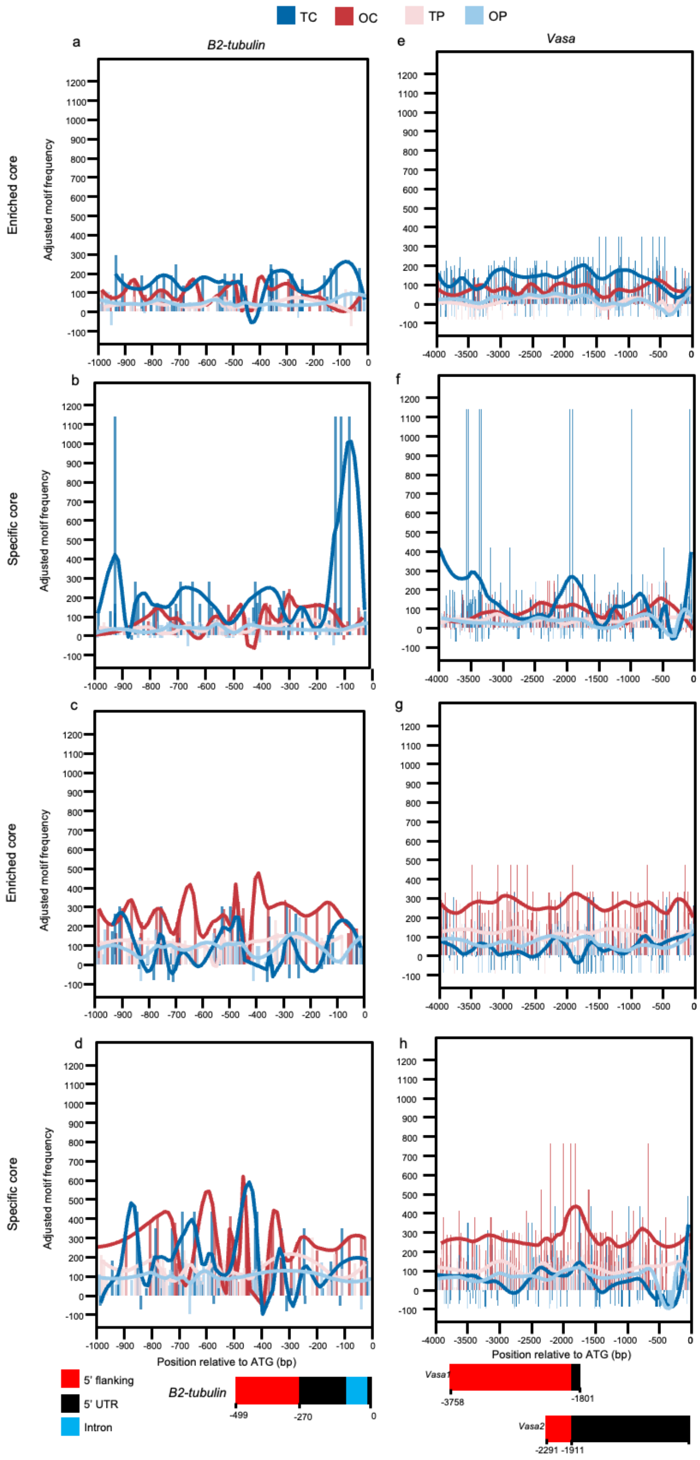

Motif frequency in the -1000 bp region upstream of the *β2-tubulin* gene adjusted by **a, b** testis regression coefficient and **c, d** ovary regression coefficient, and the -4000 bp region upstream of the *vasa* gene

adjusted by the **e, f** testis regression coefficient and **g, h** the ovary regression coefficient. The top plots (a, c, e, g) represent enriched motifs, and the bottom plots represent (b, d, f, h) specific motifs. Coloured bars and smoothing lines within the plots are indicative of the motif category based on the regression analysis (Fig. 1). Smoothing lines have been imposed using a loess smoothing method to highlight local trends for each motif category based on their frequency adjusted by testis (top graphs) or ovary (bottom graphs) regression coefficient. Putative core (-100 bp to +30 bp) and proximal (-500 bp to -101 bp) are indicated by vertical dotted lines. Horizontal bars below the plots represent the annotated regions of previously characterised *β2-tubulin* and *vasa* promoters, with promoter 5' flanking regions indicated in red, 5' UTR in black, and blue indicates the intron present within the *β2-tubulin* 5' UTR.

Supplementary Fig 4: *AgTeAc-1* chromosome enrichment, species conservation, and gene ontology enrichment.

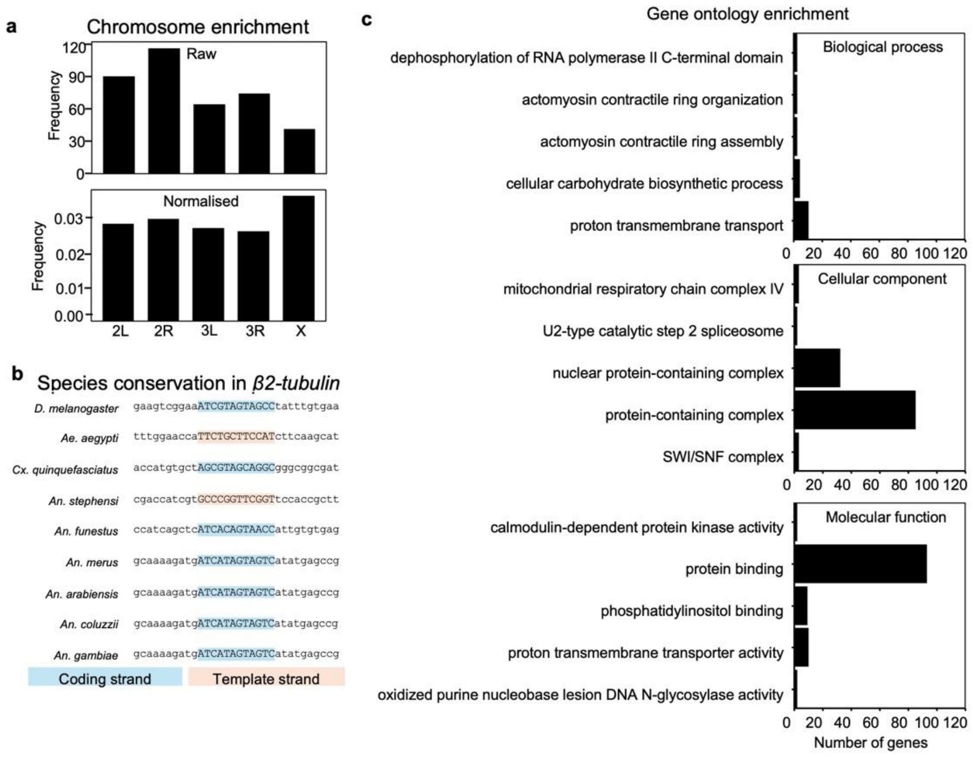

a Raw chromosome enrichment of genes containing the *AgTeAc-1* motif, and the normalised count by the number of genes on each chromosome. **b** *AgTeAc-1* motif found closest to the TSS using FIMO search for the motif in the β2-tubulin orthologs in the reference genomes of the listed organisms, which are ordered in phylogenetic distance. Sequences are listed in 5’ to 3’ with matches on the coding strand highlighted in blue, and matches on the template strand highlighted in orange, 10 nt flanking each side of the motif are also shown. **c** Gene ontology enrichment for genes containing the *AgTeAc-1* motif found using VectorBase.

Supplementary Fig 5: Schematic of validation of intact GFP cassette within transgenic lines.

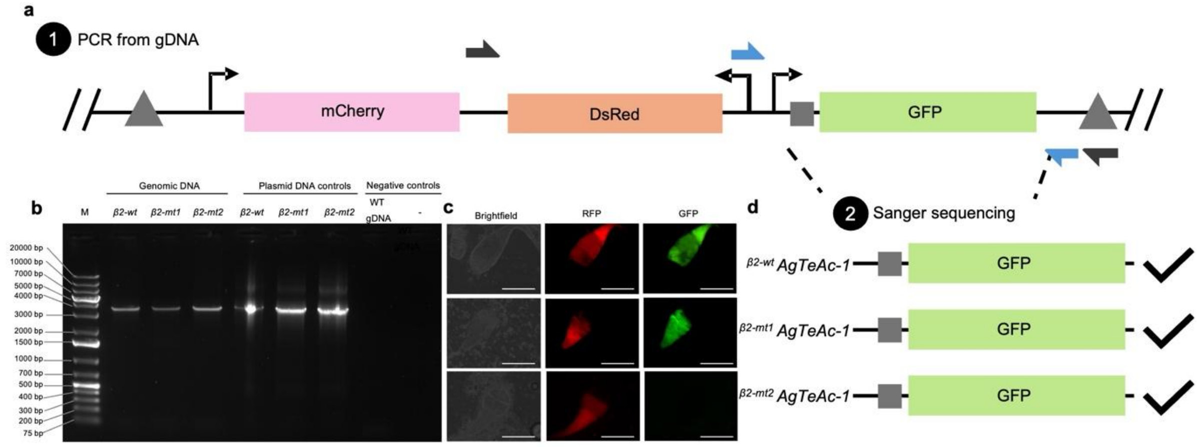

a Schematic of dual reporter construct within genome, and position of the primers (dark grey arrows) used for PCR and primers (blue arrows) used for Sanger sequencing to confirm presence of cassette. **b** Amplification of cassette from (left) genomic DNA from individual male mosquitoes from each transgenic line, (middle) plasmid DNA controls, and (right) negative controls (wild-type genomic DNA and no DNA). **c** Fluorescence imaging of dissected mosquito testis from pupae of corresponding transgenic males used to extract gDNA for downstream PCR and Sanger sequencing, carrying the following constructs: (top) $^{β2\text{-}wt}$*AgTeAc-1*, (middle) $^{β2\text{-}mt1}$*AgTeAc-1*, and (bottom) $^{β2\text{-}mt2}$*AgTeAc-1*. The white scale bar represents a 200 μm distance. Red/green look up tables have been applied to the respective RFP and GFP images. **d** Schematic representing confirmation that the mutated motif and GFP cassettes have been confirmed via PCR and Sanger sequencing for each transgenic line.

Supplementary Fig 6: Unedited gel image of GFP cassette testing. An unedited, uncropped version of the PCR gel image as included in Supp. Fig 5.

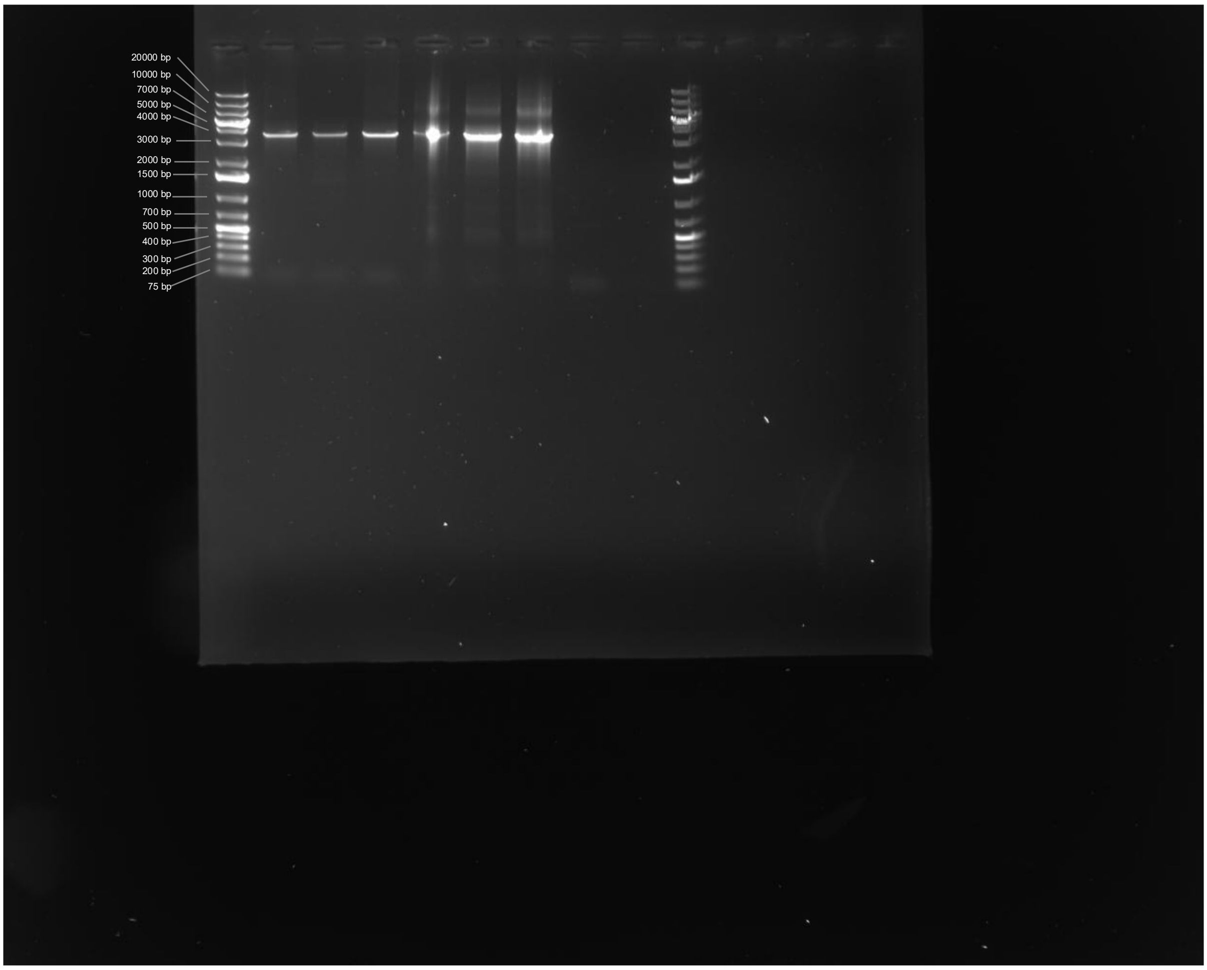

Supplement: Supplementary file 1 — Supplementary Information [file 42003_2026_10117_MOESM1_ESM.pdf]
