# Supplementary material for: Decoding cis-regulatory elements in the germline of the human malaria vector Anopheles gambiae
Source: Commun Biol. 2026 May 2;9:917. doi: 10.1038/s42003-026-10117-y (PMC13342555; doi:10.1038/s42003-026-10117-y)
Supplement: Supplementary file 2 — Description of Additional Supplementary Files [file 42003_2026_10117_MOESM2_ESM.docx]

**Description of Additional Supplementary File**

File name: Supplementary Data 1
Description: Genes used in this study and their respective tissue specificity data. Gene IDs used are shown in the first column, and their corresponding UTR lengths, and tissue-specificity data are shown in columns B-F.

File name: Supplementary Data 2
Description: Regression values for each motif. Sheets are split by the designated motif category, with the origin, PWM, and respective regression coefficients in the columns.

File name: Supplementary Data 3
Description: Matrices of motif occurrences within respective core or proximal promoters by category.

File name: Supplementary Data 4
Description: Analysed FIMO results for the β2-tubulin and vasa promoter profiling.

File name: Supplementary Data 5
Description: Raw image data for testis dissections. Sheets are split by transgenic testis dissected, with raw data and analysis data included for each testis.
