# Supplementary material for: Decoding cis-regulatory elements in the germline of the human malaria vector Anopheles gambiae
Source: Commun Biol. 2026 May 2;9:917. doi: 10.1038/s42003-026-10117-y (PMC13342555; doi:10.1038/s42003-026-10117-y)
Supplement: Supplementary file 9 — Transparent Peer Review file [file 42003_2026_10117_MOESM9_ESM.pdf]

nature portfolio

Peer Review File

Decoding cis-regulatory elements in the germline of the human malaria vector Anopheles gambiae

Corresponding Author: Dr Roberto Galizi

This file contains all reviewer reports in order by version, followed by all author rebuttals in order by version.

Version 0:

Reviewer comments:

Reviewer #1

(Remarks to the Author)
Understanding the basis of transcriptional regulation remains one of the frontiers in modern biology. In this manuscript, the authors describe a comparative bioinformatics attempt to decipher sequence motifs required for correct transcription in germline cells of a mosquito Anopheles gambiae. The approach and the findings appear to be a interesting step into that still very enigmatic biological frontier, thus, may be of interest to a wider audience.

However, there is one major issue with the presented data that must be addressed before publication is considered.
The major value of the study hinges upon validation of one motif enriched in the testis-expressed genes and found in the promoter region of the β2-tubulin gene expressed exclusively during spermatogenesis. To validate the motif, the authors used a motif mutational assay. They generated piggyBac transposition constructs, each containing two fluorescent reporters, whose expression was driven by independent β2-tubulin promoters, and used piggyBac transposition to generate one transgenic line per construct. In the transgenic line with wild-type (non-modified) β2-tubulin promoters, expression of both fluorescent markers (DsRed and GFP) was observed. In a line with one non-modified promoter (driving DsRed expression), and the other promoter (driving GFP expression) modified by substitution of conserved nucleotides within the motif, only red fluorescence was detected. That observation led the authors to an inference that the motif is necessary for correct activity of the β2-tubulin promoter in the testis. This may likely be the case. However, there is at least one alternative potential explanation for the lack of GFP expression in that line that has not been tested in this study.

During transposition, the piggyBac transposase (which in transgenesis experiments is provided in a helper plasmid) normally uses the transposon arms located on the flanks of the piggyBac transposition construct, to insert the construct into the genome, leaving the TATA dinucleotide repeat on both ends of the insertion. However, the transposition may occur not through this canonical process, and then, the construct plasmid, or a portion of the plasmid can be incorporated into the genome. If non-canonical transposition took place in this study, a portion of the plasmid with a truncated GFP cassette could have been inserted, resulting in a lack of green fluorescence in the affected line.

Therefore, the authors must demostrate that, in the transgenic line lacking GFP signal, the GFP cassette remains intact – sequence data of a preferably single PCR product (from genomic DNA) covering the whole GFP expression cassette would suffice. Only then, could they claim with large dose of likelihood (not certainty, though) that the lack of GFP is linked to the sequence modification within the conserved motif. Not certainty, because only a single transgenic line has been tested, and it remains possible (although remotely, considering expression from the neighboring DsRed cassette) that silencing of GFP might be related to the positional effect (location of the construct insertion in the genome).

Minor issues:

The manuscript is speculative in places.
Drawing a parallel between presence of repeats in putative repressor motifs and in satellite DNA found in heterochromatic regions, and implying the same silencing epigenetic mechanism operating in both cases seems unwarranted. There is qualitative difference between the two. Repeats in putative repressor motifs in question are very short, and taking into accout both their sequence and length, scattered in numerous locations in the genome. The heterochromatic minisatellite repeats are present in large blocks running in hundreds or thousands of head-to-tail, tandemly arranged copies; their sequences are known to undergo methylation leading to chromatin condensation and silencing of chromosomal blocks. If the authors know

of a published evidence, that these specific putative repressor repeats are methylated, they should present it. Otherwise, the idea remains rather speculative, which should be avoided in publications.

Further examples of speculation can be found in l. 580-583.

The authors should modulate the hype about the applicability of their approach to other systems/tissues. In their study, they focused on genes expressed in the gonads, primarily in testis, and used putative promoter regions of the top 100 most highly expressed genes to search for common motifs. Testis is a unique tissue with a highly complex transcriptome; for example, in Drosophila, it is derived from thousands of genes not expressed elsewhere. From that perspective, compared to other tissues, it may be easier to identify spermatogenesis-, or more generally, germline-specific motifs based on bioinformatics search alone.

p. 179. Golden Gate – use capital letters.

p. 203-204. Obviously, larvae cannot be used in crosses and produce progeny, as written. If anything, F0 individuals that in the larval stage showed transient DsRed expression were crossed.

Most of the first paragraph of the Results seems to belong in Methods.

p. 281-283. For the sentence starting with 'The biased representation …' a supporting reference should be added.

p. 511-512. The text mentions 2 single-nucleotide changes in β2-mt1AgTeAc-1 plasmid. Fig 7D shows substitution of 3 nucleotides.

Reviewer #2

(Remarks to the Author)
Using previously published genomic datasets, the authors describe a computational approach to identify ranked lists of cis-regulatory elements. Motif distribution was analyzed within putative promoter regions, along with copy number correlations and genome-wide distribution. Specific germline activators and repressors were identified, including a testis-specific activator that was further investigated. Transgenic An. gambiae lines were generated with a dual-reporter design incorporating this activator within a known promoter, using three motif variants, and quantitative data were collected to support the hypothesis.

This study aimed to establish a methodology for identifying cis-regulatory elements in the germline of An. gambiae. The approach involved analysing previously published datasets to define nucleotide motifs acting as positive or negative effectors of germline gene expression, using bioinformatics with appropriate controls.
Key findings include motif identification, their relative abundance and positions within candidate genes, and subsequent analysis. These results should increase confidence that in vivo studies will yield predictable and controlled expression profiles in genetically modified organisms. The study impacts the field in at least two ways: (1) providing species-specific data for gene drive and related research, and (2) offering a replicable methodology for other non-model species of interest, such as disease vectors or agricultural pests.
The paper demonstrates strong technical rigor, and conclusions are well supported by the data. I have no major or minor concerns; the study design, analyses, data, referencing, and interpretation are sound.
I am not competent or confident regarding the statistical calculations within the text and therefore have not reviewed them.

The authors clearly demonstrate a new technique for identifying CREs using published datasets. In vivo validation strengthens the evidence that this method can identify CRE candidates. The testis-enriched CRE investigation confirms the approach's validity. However, there are points that merit consideration:
1. The use of a single transgenic line does not eliminate effects of potential positional effects. Ideally, at least three independent lines should be generated to mitigate this concern. Readers assume the line is a canonical single insertion with normal Mendelian inheritance and no fitness effects—adding this information would reassure that observed effects stem from nucleotide variation rather than insertion position.
2. The study could be enhanced by including in vivo data for an ovary-enriched CRE and/or repressor motifs. While generating additional transgenics is time-consuming, such data would strengthen the study's impact and confirm its broader applicability.
3. The study would be greatly enhanced by analysis using a different non-model organism data set, though again, validation of the findings would take a great amount of effort.

Reviewer #3

(Remarks to the Author)
Title: 'Decoding cis-1 regulatory elements in the germline of the human malaria vector Anopheles gambiae'

Significant progress in regulatory genome of Anopheles gambiae is underway specially germline (testis-biased or ovary-biased genes)-specific regulatory elements, such as the vasa2 and zpg promoters, which is providing important and crucial

information for developing new mosquito control strategies.

In the present paper, the authors conclude that the putative CREs and downstream analysis demonstrate endogenous promoter elements that exhibit less leaky expression compared to current gene drive promoters, which is very promising. The work is significant in the current gene-drive control strategies.

The paper is informative, well-structured and well-written.
• The Objective of the study is meaningful and quite significant in current vector control scenario.
• Introduction of the paper is written clearly referring genetic control strategies against Anopheles stephensi and Anopheles gambiae using promoters in homing gene drives with appropriate citations.
• The Methodology is specific and appropriate.
• Linear regression is performed to correlate with actual gene expression levels helping to categorise them as putative activators or repressors, which is appropriate. Motif distribution analysis is also quite appropriate. Statistical analyses followed by the authors are justified.
• Results are explained section wise with clarity and gave new information.
All the text Figures and Supplementary materials are good.
• Discussion part is well-explained with proper and relevant citations.
• Conclusion is specific, concise, significant and fulfil the main objectives of the study.
• Bibliography is well-structured and properly selected.

It is overall a good and informative paper and it can be accepted for publication in this esteemed journal.

Version 1:

Reviewer comments:

Reviewer #1

(Remarks to the Author)
Generally, I am satisfied with the revised material. However, there are still two points that, in my opinion, require attention.

The text added in lines 522–528 needs clarification. Readers may not understand what the authors mean by “mixed transgenic progenies.” This becomes clearer only after reading the response to the reviewer’s comment, where the authors explain that, based on their prior experience with piggyBac transformation, they expected more than one independent insertion among the analyzed transgenic individuals. While this appears to be a plausible argument, it would be prudent to explicitly state in the manuscript that this was an expectation rather than a verified result.

The sentence added in lines 545-8 seems to lack logical rigour. By verifying the integrity of the tubulin::GFP cassette in the genomes of transgenic individuals, the authors can only confirm that the loss of EGFP expression was not caused by truncation or deletion of the expression construct resulting from non-canonical piggyBac transposition. They could also cite some relevant papers describing non-canonical transposition events in mosquitoes (e.g., doi: 10.1007/s11248-004-6067-2, doi.org/10.1038/hdy.2016.57).

Reviewer #2

(Remarks to the Author)
Following the revised manuscript, I am happy to recommend this paper for publication with no additional changes.

Open Access This Peer Review File is licensed under a Creative Commons Attribution 4.0 International License, which permits use, sharing, adaptation, distribution and reproduction in any medium or format, as long as you give appropriate credit to the original author(s) and the source, provide a link to the Creative Commons license, and indicate if changes were made.
In cases where reviewers are anonymous, credit should be given to 'Anonymous Referee' and the source.
The images or other third party material in this Peer Review File are included in the article’s Creative Commons license, unless indicated otherwise in a credit line to the material. If material is not included in the article’s Creative Commons license and your intended use is not permitted by statutory regulation or exceeds the permitted use, you will need to obtain permission directly from the copyright holder.
To view a copy of this license, visit https://creativecommons.org/licenses/by/4.0/

Response to Reviewers

We thank the reviewers for their thoughtful assessment and constructive feedback. We have carefully considered all comments and revised the manuscript accordingly. Below, we provide a detailed, point-by-point response to the reviewers' comments, outlining the changes implemented where applicable and clarifying how these revisions have strengthened the work.

Reviewer #1 (Remarks to the Author):

Understanding the basis of transcriptional regulation remains one of the frontiers in modern biology. In this manuscript, the authors describe a comparative bioinformatics attempt to decipher sequence motifs required for correct transcription in germline cells of a mosquito Anopheles gambiae. The approach and the findings appear to be a interesting step into that still very enigmatic biological frontier, thus, may be of interest to a wider audience.

However, there is one major issue with the presented data that must be addressed before publication is considered.
The major value of the study hinges upon validation of one motif enriched in the testis-expressed genes and found in the promoter region of the β2-tubulin gene expressed exclusively during spermatogenesis. To validate the motif, the authors used a motif mutational assay. They generated piggyBac transposition constructs, each containing two fluorescent reporters, whose expression was driven by independent β2-tubulin promoters, and used piggyBac transposition to generate one transgenic line per construct. In the transgenic line with wild-type (non-modified) β2-tubulin promoters, expression of both fluorescent markers (DsRed and GFP) was observed. In a line with one non-modified promoter (driving DsRed expression), and the other promoter (driving GFP expression) modified by substitution of conserved nucleotides within the motif, only red fluorescence was detected. That observation led the authors to an inference that the motif is necessary for correct activity of the β2-tubulin promoter in the testis. This may likely be the case. However, there is at least one alternative potential explanation for the lack of GFP expression in that line that has not been tested in this study.

During transposition, the piggyBac transposase (which in transgenesis experiments is provided in a helper plasmid) normally uses the transposon arms located on the flanks of the piggyBac transposition construct, to insert the construct into the genome, leaving the TATA dinucleotide repeat on both ends of the insertion. However, the transposition may occur not through this canonical process, and then, the construct plasmid, or a portion of the plasmid can be incorporated into the genome. If non-canonical transposition took place in this study, a portion of the plasmid with a truncated GFP cassette could have been inserted, resulting in a lack of green fluorescence in the affected line.

Therefore, the authors must demostrate that, in the transgenic line lacking GFP signal, the GFP cassette remains intact – sequence data of a preferably single PCR product (from genomic DNA) covering the whole GFP expression cassette would suffice. Only then, could they claim with large dose of likelihood (not certainty, though) that the lack of GFP is linked to the sequence modification within the conserved motif. Not certainty, because only a single transgenic line has been tested, and it remains possible (although remotely, considering expression from the neighboring DsRed cassette) that silencing of GFP might be related to the positional effect (location of the construct insertion in the genome).

We thank the reviewer for their appreciation of our approach, their thoughtful comments, and for highlighting this important point to further strengthen the in vivo validation results.
Although non-canonical piggyBac transposition events have been reported in other organisms at very low frequency, these typically involve integration at non-TTAA sites (e.g. doi: 10.1128/MCB.00670-12) or single-ended transposition (doi: 10.1371/journal.pgen.1004103), which can result in insertion of the entire plasmid, including backbone sequences outside the piggyBac terminal inverted repeats. We are not aware of any reports describing truncation of sequences internal to the piggyBac-flanked cassette within the plasmid, and this aligns with our extensive experience using piggyBac systems in *Anopheles*.
Nonetheless, to directly address the concern regarding the integrity of the GFP cassette in the transgenic line lacking GFP expression, we performed PCR and Sanger sequencing on gDNA from individual transgenic insects carrying either the wild-type or a mutated version of the AgTeAc-1 motif within the *β2-tubulin* promoter driving GFP. The same individuals were also assessed for GFP expression in the testis to link each genomic sequence with the corresponding expression phenotype. These analyses confirm the presence and full integrity of the entire *β2::GFP::terminator* cassette for all three constructs tested, as well as correspondence between the AgTeAc-1 motif genotype (wild-type, mutant 1, or mutant 2) and the observed GFP expression phenotype. Together, these results demonstrate that the loss of GFP expression is attributable to the motif modification rather than to any potential disruption of the GFP cassette.
These new data have been added as Supplementary Fig. 5, and a concise summary has been incorporated into the revised manuscript (lines 545-548 in the marked-up version of the manuscript).

Minor issues:
The manuscript is speculative in places.
Drawing a parallel between presence of repeats in putative repressor motifs and in satellite DNA found in heterochromatic regions, and implying the same silencing epigenetic mechanism operating in both cases seems unwarranted. There is qualitative difference between the two. Repeats in putative repressor motifs in question are very short, and taking into accout both their sequence and length, scattered in numerous locations in the genome. The heterochromatic minisatellite repeats are present in large blocks running in hundreds or thousands of head-to-tail, tandemly arranged copies; their sequences are known to undergo methylation leading to chromatin condensation

and silencing of chromosomal blocks. If the authors know of a published evidence, that these specific putative repressor repeats are methylated, they should present it. Otherwise, the idea remains rather speculative, which should be avoided in publications.
Further examples of speculation can be found in l. 580-583.

Our intention was not to imply that the putative repressor motifs identified in our analysis are subjected to the same epigenetic silencing mechanisms as heterochromatic satellite repeats, which we do not mention or directly correlate in the manuscript. Instead, our aim is to highlight the shared features we observe among motifs with significantly negative regression coefficients for germline expression, which we refer to as putative "repressors". These include:

1. Repetitive structures, in some cases comparable in length to microsatellites or short tandem repeats (Fig. 1 and Supplementary Data 2), although we refrain from drawing a direct connection without further investigation.
2. A strong association between their copy numbers and transcriptional inactivity in the germline (Fig. 3).
3. Enrichment on the X chromosome (Fig. 4), consistent with our previous observations in Taxiarchi et al. 2019 (doi.org/10.1038/s41598-019-51181-1) and Page et al. 2023 (doi.org/10.1038/s42003-023-05224-z) that the X chromosome undergoes broad transcriptional inactivation in the germline, in line with MSCI-associated epigenetic silencing.

In the manuscript we explicitly state that "some of the repressor motifs could act as targets of epigenetic silencing", and we further emphasise that "it is impossible to pinpoint the specific function of each motif without in vivo functional validation". Such experiments will ultimately be required to determine whether these motifs participate directly in epigenetic repression or act through alternative regulatory mechanisms. Although evidence for DNA methylation in *Anopheles* mosquitoes during germline development remains limited, we note that one of our strongest 'repressor' candidates contains CA repeats (Fig 1f). In mammals, CA-repeats have been shown to acquire methylation marks e.g. Papin et al. 2017 (doi:10.1101/gr.213983.116), and even short CA arrays (as few as five repeats) can undergo methylation-dependent modulation of protein binding in neuronal contexts Ibrahim et al. 2021 (doi:10.1126/science.abd5581). While these findings derive from mammalian systems, they demonstrate that CA-repeat–associated methylation can modulate regulatory interactions. This offers a conceptual parallel for how similar motifs could function as epigenetic targets in *Anopheles* if methylation-like pathways are indeed active, a scenario that has yet to be empirically validated. Because this question falls outside the remit of the current work, we avoid advancing any direct mechanistic interpretations that might be viewed as speculative.
The passage at lines at 614-618 (previously lines 580–583) has been updated to improve clarity on this point and reads as follows:
"Together, these results provide a first-of-its-kind classification of putative germline CREs in the mosquito germline and offer early indications of how they may cooperate in regulating gene expression. Definitive mechanistic interpretation of the functions of individual motifs will nonetheless require further investigation and targeted functional validation".

The authors should modulate the hype about the applicability of their approach to other systems/tissues. In their study, they focused on genes expressed in the gonads, primarily in testis, and used putative promoter regions of the top 100 most highly expressed genes to search for common motifs. Testis is a unique tissue with a highly complex transcriptome; for example, in Drosophila, it is derived from thousands of genes not expressed elsewhere. From that perspective, compared to other tissues, it may be easier to identify spermatogenesis-, or more generally, germline-specific motifs based on bioinformatics search alone.

Our intention was not to overstate the broad applicability of our approach, but to clarify the framework through which it can be extended. The computational pipeline is designed to identify and rank putative CREs enriched across a defined set of co-expressed genes, even when that set is relatively small (e.g., the 100 genes used in our study). Consequently, any gene set with shared regulatory logic and enrichment for relevant CREs should, in principle, be suitable for this analysis. As with any CRE-discovery method, however, in vivo functional validation remains essential to determine predictive accuracy across tissues, cell types, or species.
At the same time, although performance across species and tissues will naturally vary, the relevance within mosquito species—particularly in the germline—is substantial. Our study provides the first nucleotide-level deconstruction of germline promoters, establishing a foundation for future promoter design with the potential to advance genetic control strategies not only in *Anopheles* but also in other major pest and vector species, where promoter performance in gene-drive constructs often differs markedly and is generally lower. We have edited the final section of the conclusion (lines 672-689) to make this aspect clearer.

p. 179. Golden Gate – use capital letters.

Corrected.

p. 203-204. Obviously, larvae cannot be used in crosses and produce progeny, as written. If anything, F0 individuals that in the larval stage showed transient DsRed expression were crossed.

Corrected.

Most of the first paragraph of the Results seems to belong in Methods.

We appreciate the reviewer's observation. However, given the novelty of our approach and the feedback received from colleagues who reviewed an early draft of the manuscript, we felt it was important to introduce the key technical aspects at the start of the Results section. Providing this context up front helps readers understand the rationale behind critical methodological decisions, such as tissue selection and promoter region choice, which underpins the subsequent motif categorisation and interpretation of the analyses that follow.

p. 281-283. For the sentence starting with 'The biased representation …' a supporting reference should be added.

References added.

p. 511-512. The text mentions 2 single-nucleotide changes in β2-mt1AgTeAc-1 plasmid. Fig 7D shows substitution of 3 nucleotides.

Corrected.

Reviewer #2 (Remarks to the Author):

Using previously published genomic datasets, the authors describe a computational approach to identify ranked lists of cis-regulatory elements. Motif distribution was analyzed within putative promoter regions, along with copy number correlations and genome-wide distribution. Specific germline activators and repressors were identified, including a testis-specific activator that was further investigated. Transgenic An. gambiae lines were generated with a dual-reporter design incorporating this activator within a known promoter, using three motif variants, and quantitative data were collected to support the hypothesis.

This study aimed to establish a methodology for identifying cis-regulatory elements in the germline of An. gambiae. The approach involved analysing previously published datasets to define nucleotide motifs acting as positive or negative effectors of germline gene expression, using bioinformatics with appropriate controls.
Key findings include motif identification, their relative abundance and positions within candidate genes, and subsequent analysis. These results should increase confidence that in vivo studies will yield predictable and controlled expression profiles in genetically modified organisms. The study impacts the field in at least two ways: (1) providing species-specific data for gene drive and related research, and (2) offering a replicable methodology for other non-model species of interest, such as disease vectors or agricultural pests.
The paper demonstrates strong technical rigor, and conclusions are well supported by the data. I have no major or minor concerns; the study design, analyses, data, referencing, and interpretation are sound.
I am not competent or confident regarding the statistical calculations within the text and therefore have not reviewed them.

The authors clearly demonstrate a new technique for identifying CREs using published datasets. In vivo validation strengthens the evidence that this method can identify CRE candidates. The testis-enriched CRE investigation confirms the approach's validity. However, there are points that merit consideration:
1. The use of a single transgenic line does not eliminate effects of potential positional effects. Ideally, at least three independent lines should be generated to mitigate this concern. Readers assume the line is a canonical single insertion with normal Mendelian inheritance and no fitness effects—adding this information would reassure that

observed effects stem from nucleotide variation rather than insertion position.
2. The study could be enhanced by including in vivo data for an ovary-enriched CRE and/or repressor motifs. While generating additional transgenics is time-consuming, such data would strengthen the study's impact and confirm its broader applicability.
3. The study would be greatly enhanced by analysis using a different non-model organism data set, though again, validation of the findings would take a great amount of effort.

We thank Reviewer 2 for their thorough evaluation and positive feedback. Our responses to the points raised are provided below:
1) We agree with the reviewer's concern regarding positional effects, which would indeed introduce stochastic bias arising from genomic position-dependent and batch- or inter-individual-specific variation in expression, and therefore necessitate analysis of multiple insertions for robust interpretation of reporter activity. To mitigate this, in our study we have adopted a dual-reporter assay design in which a wild-type copy of the β2-tubulin::mCherry marker (RFP) is used for internal normalisation of EGFP expression from wild-type or CRE-mutant constructs (see Fig. 7). This configuration allows us to analyse directly mixed G2 progenies and further reduces the time and effort that would otherwise be required for isolating and characterising individual insertions.
Based on our previous experience with similar pBac-mediated transformations and subsequent crosses (see doi: 10.1038/ncomms4977, Supplementary Table 1), and considering only the independent insertions reported there (likely an underestimation), we estimate that an average of approximately four independent insertions is typically obtained from a comparable number of injected embryos and transient crosses. This suggests that, with the approach used here, we can analyse more than one insertion per construct while substantially reducing the time and resources required for this type of analysis. We have updated the section introducing the reporter assay to clarify this point (lines 522-528 in the marked-up version of the manuscript).
2) We agree with the reviewer that additional in vivo testing would further strengthen the demonstration of broader applicability. However, as acknowledged in the manuscript, the current study is constrained by the inherently low throughput- nature of the in vivo testing strategy. The use of fluorescent markers, while providing a practical analytical solution, is only feasible for promoters capable of driving sufficiently strong reporter expression. This necessitates prior validation of promoter strength, which remains very limited in *Anopheles gambiae*. For example, our recent attempt to use the ovary and -testis expressed- *zpg* promoter did not yield adequate reporter expression, even when using the wild-type promoter sequence. As we also note, future work will require higher throughput- functional characterisation, such as the use of massively parallel reporter assays (MPRAs), which are not yet available for germline specific- characterisation. We have revised and integrated the penultimate paragraph of the discussion to clarify this point more explicitly (lines 657-671).
3) We appreciate the reviewer's suggestion to extend our analysis to an additional non-model organism. While such work would indeed be valuable, it would require experimental validation of CRE activity in the new species, representing a substantial effort beyond the scope of this study. Since posting the preprint, our approach has already generated multiple collaboration requests to apply these methods to other systems, and we expect the framework and resources provided here to facilitate such

extensions. We therefore view this study as a foundation for broader comparative work, while noting that performing these additional analyses ourselves is not feasible within the scope of the current work. Additional details regarding potential future extensions of this work are now included in the final paragraph of the discussion (lines 672-689).

Reviewer #3 (Remarks to the Author):

Title: 'Decoding cis-1 regulatory elements in the germline of the human malaria vector Anopheles gambiae'

Significant progress in regulatory genome of Anopheles gambiae is underway specially germline (testis-biased or ovary-biased genes)-specific regulatory elements, such as the vasa2 and zpg promoters, which is providing important and crucial information for developing new mosquito control strategies.

In the present paper, the authors conclude that the putative CREs and downstream analysis demonstrate endogenous promoter elements that exhibit less leaky expression compared to current gene drive promoters, which is very promising. The work is significant in the current gene-drive control strategies.

The paper is informative, well-structured and well-written.
• The Objective of the study is meaningful and quite significant in current vector control scenario.
• Introduction of the paper is written clearly referring genetic control strategies against Anopheles stephensi and Anopheles gambiae using promoters in homing gene drives with appropriate citations.
• The Methodology is specific and appropriate.
• Linear regression is performed to correlate with actual gene expression levels helping to categorise them as putative activators or repressors, which is appropriate. Motif distribution analysis is also quite appropriate. Statistical analyses followed by the authors are justified.
• Results are explained section wise with clarity and gave new information.
All the text Figures and Supplementary materials are good.
• Discussion part is well-explained with proper and relevant citations.
• Conclusion is specific, concise, significant and fulfil the main objectives of the study.
• Bibliography is well-structured and properly selected.

It is overall a good and informative paper and it can be accepted for publication in this esteemed journal.

We thank Reviewer 3 for their positive evaluation of the manuscript and appreciate their supportive comments.

General editorial comments

We request that the experiments mentioned by Reviewers 1 and 2 to validate the transposition be conducted. We also suggest including a discussion on previous work

which uses other sources of information for identifying regulatory elements in this organism (for example, Ruiz et al 2021 NAR Genomics and Bioinformatics; Kalita et al 2025 Genes and Dev; Sieglaff et al 2009 PNAS)

In addition to incorporating new data and textual revisions in response to the reviewers' comments, we updated both the Introduction (lines 94–102 in the marked-up version of the manuscript) and Discussion (lines 573–577) to include and discuss previous work on identifying regulatory elements in *Anopheles*, as requested.

Response to referees - Chesters et al Revision 2

We thank the reviewers for their thoughtful feedback and constructive comments throughout the review process.

Reviewer #1 (Remarks to the Author):
Generally, I am satisfied with the revised material. However, there are still two points that, in my opinion, require attention.
The text added in lines 522–528 needs clarification. Readers may not understand what the authors mean by "mixed transgenic progenies." This becomes clearer only after reading the response to the reviewer's comment, where the authors explain that, based on their prior experience with piggyBac transformation, they expected more than one independent insertion among the analyzed transgenic individuals. While this appears to be a plausible argument, it would be prudent to explicitly state in the manuscript that this was an expectation rather than a verified result.

We have further clarified this point as follows: "This configuration enables direct analysis of mixed transgenic progenies, which are expected to carry a diverse range of genomic insertions based on our previous experience using the same transposase-mediated integration system [13,14], although this was not explicitly tested in the present study. The dual-reporter system we adopted therefore provides an efficient means to assess multiple integration events simultaneously, without the need to isolate and characterise each insertion individually."

The sentence added in lines 545-8 seems to lack logical rigour. By verifying the integrity of the tubulin::GFP cassette in the genomes of transgenic individuals, the authors can only confirm that the loss of EGFP expression was not caused by truncation or deletion of the expression construct resulting from non-canonical piggyBac transposition. They could also cite some relevant papers describing non-canonical transposition events in mosquitoes (e.g., doi: 10.1007/s11248-004-6067-2, doi.org/10.1038/hdy.2016.57).

In addition to our previous response and additional data we have included in the revised manuscript, we have now further clarified this point and included the suggest references as follows: "To exclude the possibility that the absence of EGFP expression could have resulted from non-canonical *piggyBac*-mediated transposition events—reported in other mosquito species [56,57] but, to the best of our knowledge, not detected in *A. gambiae* [13]—we confirmed the presence of the dual-reporter genomic insertion and the sequence integrity of the *b2-tubulin::EGFP* cassette in individual mosquitoes. These individuals were simultaneously analysed for fluorescence expression in dissected testes (Supplementary Fig. 5, Supplementary Fig. 6), further supporting that the reduction in EGFP expression was due to the mutations introduced within the *AgTeAc-1* motif".

Reviewer #2 (Remarks to the Author):
Following the revised manuscript, I am happy to recommend this paper for publication with no additional changes.
Thank you.
